# Identification of miRNAs and Their Response to Cold Stress in *Astragalus Membranaceus*

**DOI:** 10.3390/biom9050182

**Published:** 2019-05-10

**Authors:** Merhaba Abla, Huigai Sun, Zhuyun Li, Chunxiang Wei, Fei Gao, Yijun Zhou, Jinchao Feng

**Affiliations:** 1 College of Life and Environmental Sciences, Minzu University of China, Beijing 100081, China; merhaba@muc.edu.cn (M.A.); 15055031@muc.edu.cn (Z.L.); chunxiangwei2010@163.com (C.W.); zhouyijun@muc.edu.cn (Y.Z.); jchfeng@263.net (J.F.); 2 School of Pharmacology, Hebei University of Chinese Medicine, Shijiazhuang 050200, China; sunhuigai66@163.com

**Keywords:** *Astragalus membranaceus*, miRNA, cold stress, miR390

## Abstract

*Astragalus membranaceus* is an important medicinal plant widely cultivated in East Asia. MicroRNAs (miRNAs) are endogenous regulatory molecules that play essential roles in plant growth, development, and the response to environmental stresses. Cold is one of the key environmental factors affecting the yield and quality of *A. membranaceus*, and miRNAs may mediate the gene regulation network under cold stress in *A. membranaceus*. To identify miRNAs and reveal their functions in cold stress response in *A. membranaceus*, small RNA sequencing was conducted followed by bioinformatics analysis, and quantitative real time PCR (qRT-PCR) analysis was performed to profile the expression of miRNAs under cold stress. A total of 168 conserved miRNAs belonging to 34 families and 14 putative non-conserved miRNAs were identified. Many miRNA targets were predicted and these targets were involved in diversified regulatory and metabolic pathways. By using qRT-PCR, 27 miRNAs were found to be responsive to cold stress, including 4 cold stress-induced and 17 cold-repressed conserved miRNAs, and 6 cold-induced non-conserved miRNAs. These cold-responsive miRNAs probably mediate the response to cold stress by regulating development, hormone signaling, defense, redox homeostasis, and secondary metabolism in *A. membranaceus*. These cold-corresponsive miRNAs may be used as the candidate genes in further molecular breeding for improving cold tolerance of *A. membranaceus*.

## 1. Introduction

*Astragalus membranaceus* (Fisch.) Bge is a perennial flowering leguminous plant that is mainly distributed in the cool arid continental regions of the Northern Hemisphere and South America. The dry root of *A. membranaceus* is one of the most important traditional herbal medicines that has been widely used in many herbal formulations in China, Korea and other Eastern Asia countries. A variety of pharmacological activities, i.e., immunoregulatory [1], anti-inflammatory [2], anticancer [3], and antihyperglycemic [4], have been reported for the root of *A. membranaceus*. The herb has been used to treat various diseases including chronic fatigue, wounds, and anemia [5]. Especially, a compound extracted from *A. membranaceus*, TA-65, has been shown to retain antiaging activity by acting as a telomerase activator [6]. In addition, the root is also widely used as a health food supplement around the world.

As an important traditional medicinal herb, *A. membranaceus* is widely cultivated in Southeast Asia, including Inner Mongolia autonomous district, Shanxi province, Gansu province, Heilongjiang province and other regions in North China. Several studies proposed that the yield and quality of *A. membranaceus* were affected by environmental factors like soil water content, light, and temperature [7,8]. Low temperature is one of the most important environmental factors that drastically limit the worldwide plant growth and crop production. Considered that *A. membranaceus* is mainly distributed in temperate regions, the yield was inevitably negatively affected by cold injury frequently occurring in early spring and late autumn. But, on the other hand, a certain degree of cold stress might contribute to accumulation of the pharmacological active ingredients, most of which were derived from the phenylpropanoid pathway in *A. membranaceus* [7]. Thus, elucidation of the cold stress response would be beneficial to improve the cultivation techniques of *A. membranaceus* aimed at achieving high quality and high yield simultaneously.

MicroRNAs (miRNAs) are a class of single strand, endogenous non-coding small RNAs (sRNAs) that negatively modulate gene expression at the post-transcriptional levels via mRNA cleavage or translational repression in plants and animals. In higher plants, a growing body of evidence showed that miRNAs play essential roles in growth, development, and the responses to environmental stress [9]. Many cold stress-responsive miRNAs, including miR396, miR397, and miR319, have been identified in various plant species, such as wheat [10], rice [11], Arabidopsis [12], tomato [13], and *Brachypodium distachyon* [14]. Recent studies showed that miR396 positively regulated cold tolerance by repressing ethylene synthesis through reducing 1-aminocyclopropane-1carboxylic acid oxidase (ACO) transcript levels in Arabidopsis [15]. Overexpression of miR319 in rice led to enhanced cold tolerance probably via reducing the expression level of the *OsPCF5 and OsPCF8*, two TCP family transcription factors [16]. Overexpression of miR397 significantly improved plant tolerance to chilling and freezing stresses in Arabidopsis by enhancing the expression of cold-regulated C-repeat binding factors (CBFs) and the related downstream genes [17]. In brief, miRNAs have been demonstrated to play key roles in cold stress response and adaptation.

As an important regulatory factor involved in plant development and stress response, miRNA may participate in the response of *A. membranaceus* to cold stress. Although numerous miRNAs have been identified in plant species, most of them were identified from model species and crops such as *Arabidopsis thaliana* [18], *Oryza sativa*, cotton [19], alfalfa [20], *Physcomitrella patens* [21] and *Populus trichocarpa* [22]. To date, miRNAs from *A. membranaceus* have not been reported, and the expression pattern of *A. membranaceus* miRNAs in responding to cold stress conditions were still unknown. In the present study, we systematically identified the miRNA in *A. membranaceus* for the first time, predicted their target genes, and analyzed the expression patterns under low temperature stress. The present study provided important data for understanding the roles of miRNA in regulating diversified biological pathways in cold response of *A. membranaceus*.

## 2. Materials and Methods

### 2.1. Plant Material and Cold Stress Treatment

The seeds of *A. membranaceus* were collected from Erdos city, Inner Mongolia autonomous district, China. *A. membranaceus* plants (with 9–12 plants in each 30 L pot filled with the vermiculite-mixed soil) were cultured in greenhouse at 20–25 °C and 40%~50% relative humidity with 16 h of light per day and leaf and root samples of *A. membranaceus* were collected from eight-week-old plants, frozen in liquid nitrogen, and then stored at −80 °C for further RNA extraction.

For cold stress treatment, eight-week *A. membranaceus* seedlings of similar height (8–9cm) were randomly divided into 5 groups, and 4 groups of these seedlings were transferred to a growth chamber (4–5 °C, Pervical LT-36VL, Percival Scientific, Inc. Perry, IA, USA), the leaf samples were collected after 3 h, 6 h, 24 h, and 72 h. The leaf sample collected before cold treatment were used as the control.

### 2.2. Small RNA Library Construction and Sequencing

Total RNA was extracted using Trizol reagent (Invitrogen, CA, USA) from leaves and roots of *A. membranaceus* seedlings following the manufacturer’s instructions. The quantity and purity of total RNA were checked by using Bioanalyzer 2100 and RNA 6000 Nano LabChip Kit (Agilent, CA, USA) with RIN value >7.0. Approximately 1 µg of total RNA pooled from equal amount of RNA samples from leaves and roots were used to prepare small RNA library according to protocol of TruSeq Small RNA Sample Prep Kits (Illumina, San Diego, CA, USA). In brief, the process is as follows: First, the 3′ and 5′ adapters were ligated to the total RNA, then, the resulting RNA samples were used as the templates for cDNA synthesis, third, PCR was conducted to amplify the cDNA, and fourth, PCR products were purified using 6% polyacrylamide gel electrophoresis. The single-end sequencing (36 bp) was performed on an Illumina Hiseq2000 at the LC Sciences (Hangzhou, China) following the manufacturer’s protocol. Raw sequencing reads were obtained by using Illumina’s analysis software (Illumina Inc., San Diego, CA, USA).

### 2.3. Transcriptome Sequences Assembly

All available *A. membranaceus* high throughput sequencing reads (Accession number: ERR706814, SRR923811) were downloaded from NCBI SRA database (before 8 May 2017). The raw reads were first processed with Trimmomatic (v0.3) [23] and solexaQA [24], then assembled using Trinity software [25]. The parameters used for read processing using Trimmomatic were set as follows: Java -jar trimmomatic.jar PE -threads 4 -trimlog./log.txt sample1_R1.fq sample1_R2.fq -baseout sample1_clean.fq. The parameters used for Trinity assembly were: Trinity --seqType fq --max_memory 100G --left sample1_clean_R1.fq sample2_clean_R1.fq... --right sample1_clean_R2.fq sample2_clean_R2.fq... --CPU 8. The command used for solexaQA was: perl SolexaQA.pl sample*.fq -d./-illumina.

### 2.4. Identification of Conserved and Non-Conserved miRNAs

Raw reads obtained from the sequencer were processed by removing contaminant reads including those reads with 5′ primer contaminants, reads without 3′ primer, reads with poly A, and reads with length less than 18 nt. The resulting “clean reads” were used for length distribution analysis. Then, rRNAs, tRNAs, snRNAs and snoRNAs were further removed from the clean reads sequences through BLASTN search using Rfam database (http://www.sanger.ac.uk/Software/Rfam/). The remaining distinct sRNA sequences (mappable reads) were used for identification of conserved and non-conserved miRNAs from *A. membranaceus*.

The remaining distinct sequences were mapped to the *A. membranaceus* transcriptome sequences, and the potential miRNAs were identified by folding the flanking genome sequence of distinct small RNAs using the ACGT101-miR program (version 4.2) (LC Sciences, Hangzhou, China). Reads that map more than 20 times were discarded. The other parameters were set based on the criteria for annotation of plant miRNAs [26]. All predicted stem-loop precursors were checked manually and the false positive results were removed.

Among all potential candidate miRNAs, the miRNA that shows similarity (allow no more than 3 mismatches) to the sequence of known green plant miRNAs from miRBase 21.0 (http://www.mirbase.org) was classified as “conserved miRNA” (conserved miRNA with identified stem-loop precursor). The remaining potential miRNA candidates were classified as “non-conserved miRNA”.

In addition, due to the lack of genome information, the stem-loop precursors of many conserved miRNAs cannot be identified from the assembled transcriptome sequences. These conserved miRNAs were further identified by aligning the mappable reads to the miRNA database (miRBase 21) and only perfectly matched sRNA sequences with known green plant miRNAs were considered as conserved miRNA (conserved miRNA without identified stem-loop precursor).

### 2.5. qRT-PCR Analysis of miRNAs

qRT-PCR analysis was conducted using the miRcute miRNA qPCR detection kit (Tiangen, Beijing, China). Each PCR reaction was performed in a volume of 20 µL containing 10 µL of 2×miRcute miRNA Premix, 0.4 µL of each forward primer (10 μmol/L), 0.4 µL of universal reverse primer and 1 µL of reverse-transcribed cDNA from ~20 ng of total RNA. The PCR protocol was 2 min at 95 °C, 40 cycles of 95 °C for 20 s, 60 °C for 34 s. The primers that were used in the present study are listed in additional file Table S1. Then, qRT-PCR was performed on a MyiQ2 Real-Time Detection System (Bio-Rad, Hercules, CA, USA) using the SYBR Green I method, and all reactions of qRT-PCR were repeated three times for each sample. The melting curve was used to evaluate the specificity of PCR products. U6 snRNA was used as the internal control gene in qRT-PCR analysis. Gene expression data were obtained from three biological replicates and statistical significance was evaluated using a Student’s t-test analysis. The expression level of each miRNA was normalized to that of U6 and the 2^−ΔΔCt^ method was used to calculate the relative expressional levels of miRNAs [27]. We considered a variation in expression level when a difference of at least two-fold was observed with a *p* value < 0.05.

### 2.6. Target Gene Prediction of Identified miRNAs and Gene Ontology Analysis of the Targets

The potential targets of the identified *A. membranaceus* miRNAs were predicted using the psRNATarget program http://bioinfo3.noble.org/psRNATarget/ [28], and the parameters were set as follows: Number of top targets, 200; Expectation, 3; penalty for G:U pair, 0.5; penalty for other mismatches, 1; extra weight in seed region, 1.5; seed region, 2–13 nt; number of mismatches allowed in seed region, 2. Newly identified *A. membranaceus* miRNA sequences were used as custom miRNA sequences and the assemble transcriptome sequences were used as custom plant databases.

To annotate the target transcripts, blastx was performed using the sequences of target transcripts and the TAIR10 peptide database (http://www.arabidopsis.org/). We used agriGO [29] to conduct the Gene Ontology (GO) classification and enrichment analyses for the target transcripts.

### 2.7. qRT-PCR Analysis of the miRNA Targets

qRT-PCR analysis of the expression levels of miRNA targets were conducted according to the methods described previously [30]. Three independent biological replicates for each sample and four technical replicates of each biological replicate were analyzed using qRT-PCR. The expression levels of selected targets were normalized against an internal reference gene, 18S rRNA (GenBank accession number AF359594). The relative gene expression was calculated using the 2^−ΔΔCt^ method [27]. Standard deviations were calculated from three biological replicates. The primers used for qRT-PCR analyses are listed in Supplementary Table S2.

## 3. Results

### 3.1. Summary of Small RNA Library Dataset by Deep Sequencing in A. Membranaceus

To identify miRNAs in *A. membranaceus*, a small RNA (sRNA) library from pooled RNA samples of leaves and roots of *A. membranaceus* were sequenced using the high-throughput Illumina sequencing platform, which generated 9,685,427 raw reads (Table 1). After removing adaptors, low quality sequences and sequences shorter than 18 nt, 9 M sRNA clean reads with length ranging from 18–25 nt were obtained (Table 1). All raw reads were deposited at SRA database with the accession number SRR3990711. By aligning to the Rfam database, a substantial number of rRNAs, tRNAs, snRNAs, snoRNAs and other Rfam RNA were identified from the sRNA library (Table 1).

**Figure 1:**
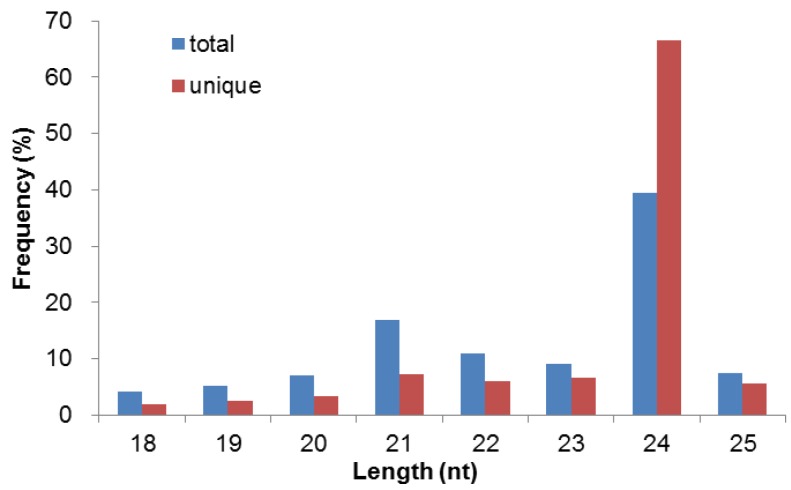
Length distribution of small RNAs in the library of *A. membranaceus*. *X*-axis, size group of small RNA; *Y*-axis, corresponding percentage of raw reads.

Among the clean reads, the number of 24-nt sequences was significantly greater than the other sequences and this group of sRNA accounted for 83.34% of the total reads (Figure 1). A total of 968,674 21-nt sRNAs (16.81%) represented the second abundant category of sequences in the sRNA library, which is the typical length of plant miRNAs. The third abundant size group of sRNAs was 22 nt (10.99%), followed by 23 nt (9.06%) and 20 nt (7.44%).

In addition, another sRNA library generated from leaf samples of *A. membranaceus* (NCBI SRA accession number SRR8929862) was also used for miRNA identification. The total number of clean reads of this sRNA library is more than 6M.

### 3.2. Identification of Conserved miRNAs and their Stem-Loop Precursors in A. Membranaceus

Since there is no genome information on *A. membranaceus*, we downloaded all available *A. membranaceus* high-throughput sequencing reads, and assembled these sequences using Trinity software. The resulting assembly contained 86,647 unigenes, ranging from 201–12,112 nt, and have an N50 of 1350 nt and an average size of 814 nt. These transcriptome sequences were used as the reference sequences for miRNA identification and miRNA target prediction.

A total of 168 distinct mature miRNA sequences were identified as conserved miRNAs (Table 2 and Table S3). The assembled transcriptome sequences of *A. membranaceus* allows us to identify 86 stem-loop precursors containing the mature forms of 69 distinct conserved miRNAs (Table 2 and Table S4). The conserved miRNAs listed in Table 2 were similar or identical to at least one of the registered miRNAs from other plant species in miRBase 21 database (http://www.mirbase.org/), and the stem-loop structures of their precursors had MFEs ranging from −100.2 to −31.50 kcal/mol with an average of −48.86 kcal/mol, and MFE/nucleotide values ranging from −0.20 to −0.59 kcal/mol/nt with an average of −0.42 kcal/mol/nt (Table S4).

The remaining 99 predicted conserved miRNAs (Table S3) were perfectly identical in sequence to at least one of the highly conserved miRNAs or legume miRNAs registered in miRBase, and the majority of these RNAs presented in considerable abundance. However, since there is no *A. membranaceus* genome information available publicly, we cannot find the corresponding stem-loop precursors for these predicted conserved miRNAs.

The conserved miRNAs were grouped into 34 miRNA gene families according to the alignment results of their mature sequences to the corresponding datasets in miRBase (Table 2 and Table S3). Many miRNA families showed significant conservation among multiple plant species, while the others exhibited less evolutionary conservation. For instance, MIR156, MIR159, MIR166, MIR169, MIR396, and MIR398, are highly conserved in a variety of plant species, whereas MIR408, MIR818, MIR828, and MIR858 were only reported in several plant species. Some other miRNA families were only reported in a couple of plant species. For example, MIR4416 was only reported in *Glycine max* [31], and MIR4415 and MIR1514 were identified only in *G. max* and *Phaseolus vulgaris* [32,33], and MIR5083 was only identified from a few plant species such as rice and barley [34].

**Figure 2:**
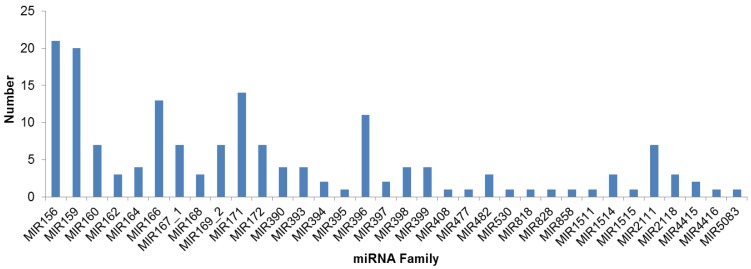
The distribution of the identified distinct miRNA sequences of each conserved miRNA family in *A. membranaceus*.

Among the 34 conserved miRNA gene families, more than 10 miRNA sequences (5p or 3p form) were identified in highly conserved miRNA family MIR156, MIR159, MIR166 and MIR171, while only one miRNA sequence was identified in some less-conserved miRNA families, for example MIR530, MIR818, MIR828, and MIR858 (Figure 2).

**Figure 3:**
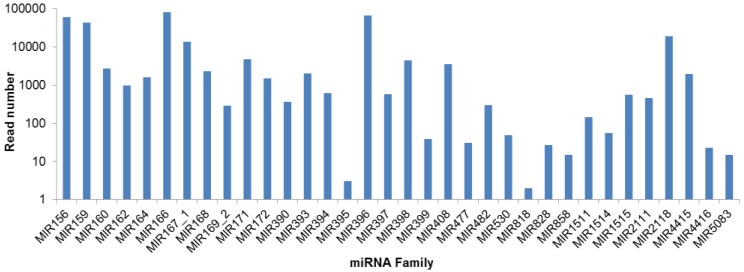
The read counts of each conserved miRNA family in *A. membranaceus*.

The abundance of each miRNA family was analyzed based on the number of reads (Figure 3). A significant difference of the read numbers among these miRNA families was observed. The MIR166 was the most abundant miRNA family in *A. membranaceus*, with a total read number of 88,223. MIR396, MIR156, MIR159, MIR2118 and MIR167_1 also exhibited very high expression level, with total read numbers more than ten thousand. While several miRNAs, i.e., MIR818, MIR395, MIR5083, MIR858, MIR4416, MIR828, MIR477, MIR399, and MIR530, exhibited very low expression level (less than 50 reeds).

### 3.3. Identification of Non-Conserved miRNAs in A. Membranaceus

**Figure 4:**
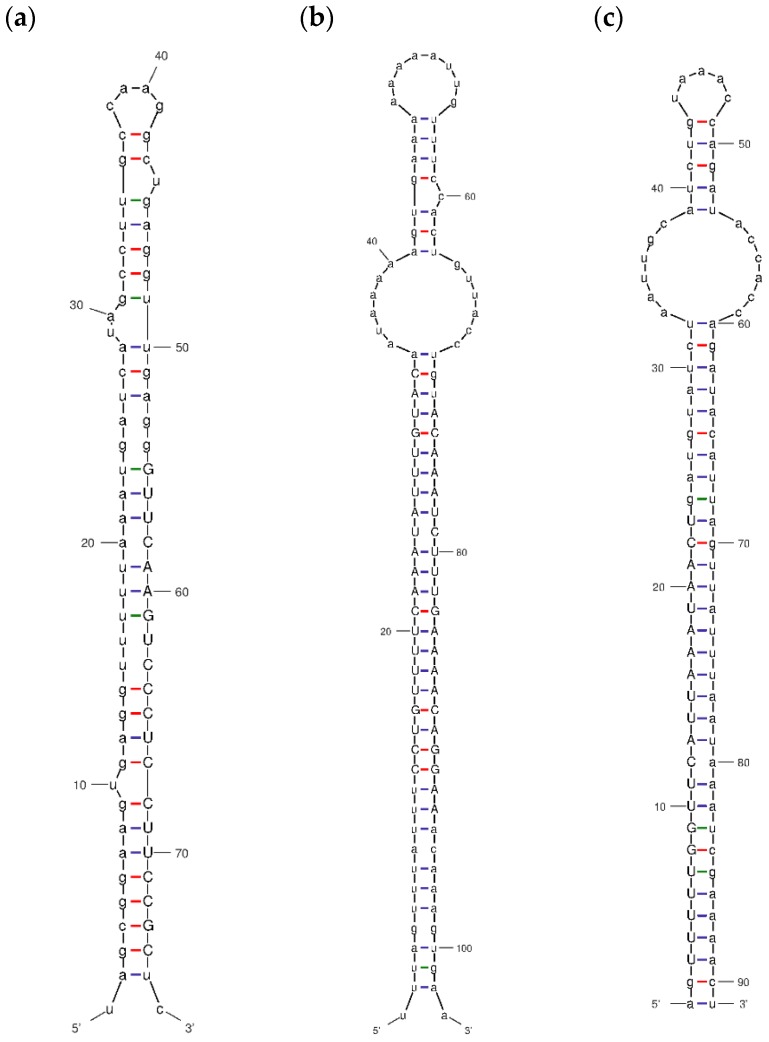
The secondary structure of the miRNA precursors of ame-miRN1 (**a**), ame-miRN8 and ame-miRN9 (**b**), and ame-miRN-14 (**c**). The mature sequences of the miRNAs were shown in uppercase. These graphs were generated by using mfold web server.

In total, 14 non-conserved miRNAs and the corresponding stem-loop precursors were identified (Table 3 and Table S5). These new miRNAs were named in the form of ame-miRN-number, e.g., ame-miRN-1, and their mature miRNA sequences were 19–23 nt in length. The predicted hairpins of their precursors had an MFE ranging from −61.60 to −33.30 kcal/mol with an average of −45.57 kcal/mol, and MFE/nucleotide values ranging from −0.19 to −0.50 kcal/mol/nt with an average of −0.33 kcal/mol/nt. The secondary structure of three miRNA precursors were shown in Figure 4.

All the non-conserved miRNA identified from *A. membranaceus* have not been reported in other plant species in the miRbase database, thus they probably represent species-specific miRNA in *A. membranaceus*. However, ame-miRN-2 was also identified from strawberry in a recent study [35], indicating ame-miRN-2 might not be a species-specific miRNA. Comparing with the conserved miRNA, the read counts of the majority of non-conserved miRNAs were very low, which was in line with the observations that non-conserved miRNAs usually expressed at a lower level than conserved miRNAs [36]. However, two miRNAs (ame-miRN2 and ame-miRN3) were found to have read numbers more than 1000. These *A. membranaceus* specific miRNA may play important roles in regulating gene expression in *A. membranaceus*.

### 3.4. Validation of the Identified miRNAs and their Expression Level in Leaves and Roots by qRT-PCR

**Figure 5:**
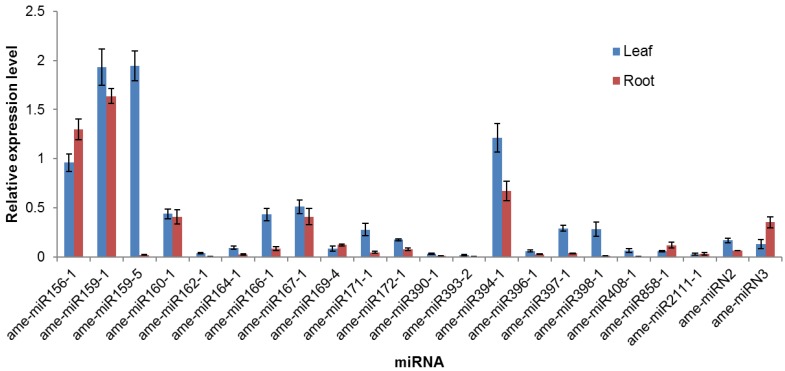
Expression of selected miRNAs in *A. membranaceus* leaves and roots. The expression level of each miRNA was normalized to that of U6. Error bars indicate SD between replicates.

To validate the existence of the identified *A. membranaceus* miRNAs, 20 conserved miRNAs and 2 non-conserved miRNAs were selected for qRT-PCR analysis. The qRT-PCR results demonstrated that all tested miRNAs were expressed in *A. membranaceus* leaves and roots (Figure 5). There was significant difference between the expression of different miRNAs and the expression levels of each miRNA varied with the tissues. In general, most of the qRT-PCR results of the high abundance miRNAs were consistent with the results from sequencing data. For example, miRNAs in family MIR156, MIR159, MIR167, MIR166, MIR394, and MIR160 were shown to express at high level by qRT-PCR. We also noticed that 14 miRNAs exhibited tissue-specific expression pattern, including ame-miR159-5, ame-miR162-1, ame-miR164-1, ame-miR166-1, ame-miR171-1, ame-miR172-1, ame-miR390-1, ame-miR393-1, ame-miR397-1, ame-miR398-1, ame-miR408-1, and ame-miRN2, which are dominantly expressed in leaves, and ame-miR858-1, and ame-miRN3, which is preferentially expressed in roots.

### 3.5. Target Prediction of A. Membranaceus miRNAs

To understand the functions of the identified *A. membranaceus* miRNAs, putative targets of these miRNAs were predicted using the psRNAtarget program [28]. Consequently, 554 and 63 putative target genes were predicted for 153 conserved and 8 non-conserved miRNAs, respectively (Supplementary Tables S6 and S7).

Based on the BLASTX alignment, more than 60% of the predicted miRNA targets were annotated by Arabidopsis peptide sequences (TAIR10). A number of targets were transcription factor genes, including squamosa promoter binding protein-like (SPL, targets of MIR156), growth regulating factors (GRF, targeted of MIR396), MYB transcription factors (targets of MIR159), TCP transcription factors (targets of MIR159), auxin response factors (ARF, targets of MIR160), NAC transcription factors (targets of MIR164), HD-Zip transcription factors (targets of MIR166), and NF-YA transcription factors (targets of MIR169). Several miRNAs of MIR159, MIR2118, and MIR482 families were predicted to target disease resistance gens such as LRR and NB-ARC domains-containing disease resistance protein genes. Most of the above miRNA-target pairs have been reported frequently in other plant species, confirming the conservation of the targets of the conserved miRNAs among different plant species.

In addition to the well-documented conserved targets, many novel targets were also identified (Tables S6 and S7). For instance, miR169-3 was found potentially to target a gene encoding high-affinity nickel-transport family protein. Although the newly-predicted miRNA-target relationship has still to be validated experimentally, these results strongly suggest that the identified *A. membranaceus* miRNAs are involved in regulation of various biological process, including morphological construction, development, and biotic and abiotic stress response.

**Figure 6:**
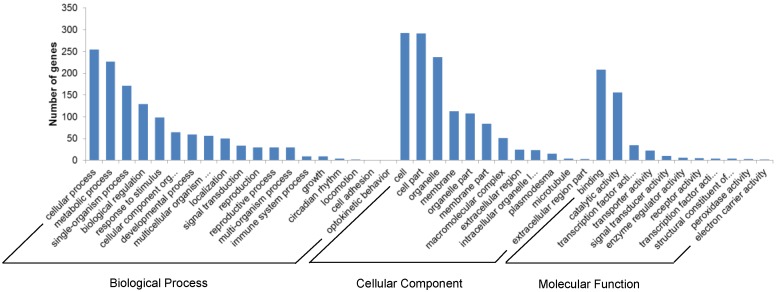
Gene ontology terms and numbers of the predicted miRNA targets.

A total of 42 GO (Gene Ontology) categories were assigned for target genes (Figure 6). Based on the biological process, the genes were classified into 19 categories of which the top three GO terms were cellular process, metabolic process, and single-organism process. In the case of molecular function, the genes were classified into 11 categories, of which they are mostly involved in binding, catalytic activity, and transcription factor activity. Based on cellular components, the genes were classified into 12 categories, of which the top three abundant were cell, cell part and, organelle. GO enrichment analysis revealed that a batch of GO terms were enriched in miRNA targets (The top ten enriched GO terms were highlighted in Supplementary Figure S1). In brief, the top 3 most over-presented GO (biological process) BP terms were macromolecule metabolic process, cellular macromolecule metabolic process, and nitrogen compound metabolic process; the top 3 GO (cellular component) CC terms were nucleus, cell part, and cell, and top 3 GO (molecular function) MF terms were transcription factor activity, cation binding, and ion binding.

Many miRNA targets encoding proteins with enzyme activity. Among these protein products, the top abundant category is hydrolases (55 genes), followed by transferases (46), oxidoreductases (17), ligases (6), isomerases (5), and lyases (4). These enzymes were involved in pathways such as protein modification (10), amino-acid biosynthesis (3), glycan metabolism (2), sulfur metabolism (2), plant hormone metabolism (2) and phenylpropanoid and flavonoid biosynthesis (2).

### 3.6. Non-Coding RNAs Targeted by miRNAs in A. Membranaceus

Many predicted miRNA targets cannot be annotated as protein coding genes and some of them may represent non-coding RNAs. PhasiRNA (tasiRNAs) is a category of secondary, phased small interfering RNAs derived from protein-coding or non-coding loci (*PHAS*) and phasiRNAs are considered as a new class of regulators of gene expression in plants [37]. Considering that phasiRNAs are mainly targeted and triggered by miRNAs, we used two phasiRNA predicting softwares, i.e., PhaseTank (v1.0) [38] and the UEA small RNA workbench (v3.2) [39] to find potential PhasiRNA in the list of miRNA targets, but no *PHAS* loci was identified from the transcriptome sequences. One of the possible reasons for this failure is the inadequate depth or coverage of the sRNA sequencing. We then manually checked the targets of the phasiRNA-triggering miRNAs, e.g., miR390, miR828 by blast alignment, and a transcript, *comp6362_c0_seq1*, was found to be a homologue of Arabidopsis *TAS3*, which is a non-coding target of miR390.

Among the four TAS gene families, *TAS3* is highly conserved in plants, while *TAS1*, *TAS2*, and *TAS4* are identified only in *Arabidopsis* and its close relatives [40]. Two miR390 complementary sites (binding site) were found in *TAS3* locus and the cleavage occurs at the downstream one, but not at the upstream one. The PhasiRNA generating region fell between the two miR390 binding sites [41].

We then mapped all sRNA sequences to the identified *A. membranaceus TAS3* (*comp6362_c0_seq1*) to find the PhasiRNAs generated from this locus, and two PhasiRNAs were identified (phasiRNAs_1: TTCTTGACCTTGTAAGACCTT, with a read number of 104, phasiRNAs_2: TTCTTGACCTTGTAAGACCTC, with a read number of 77). The targets of these phasiRNAs were further predicted using psRNAtarget software and, as expected, an *auxin response factor 2* (*ARF2*) gene was shown to be targeted by phasiRNAs_1. At the same time, a *carotenoid cleavage dioxygenase 8* (*CCD8*), an enzyme involved strigolactone biosynthetic pathway was targeted by phasiRNAs_2. Collectively, our data indicated that, PhasiRNAs derived from *AmTAS3* (*comp6362_c0_seq1*) probably involved in development by participated in auxin response and strigolactone biosynthesis in *A. membranaceus* (Figure S2).

It was noteworthy that some targets of *A. membranaceus* MIR156 were homologues of *Arabidopsis GUT15* (*AtGUT15*, AT2G18440), a long noncoding RNA [42].

### 3.7. The Expression Pattern of miRNAs in A. Membranaceus Leaves under Cold Stress

miRNAs have been reported to be involved in the cold stress response and acclimation. To identify cold stress-responsive miRNAs and reveal the dynamic expression pattern of miRNAs in *A. membranaceus* leaves during cold stress, qRT-PCR analyses were performed. For conserved miRNAs, one or two highly expressed miRNAs in each miRNA family were used for qRT-PCR analysis. For non-conserved miRNAs, six highly expressed miRNAs were selected and used for qRT-PCR analysis.

**Figure 7:**
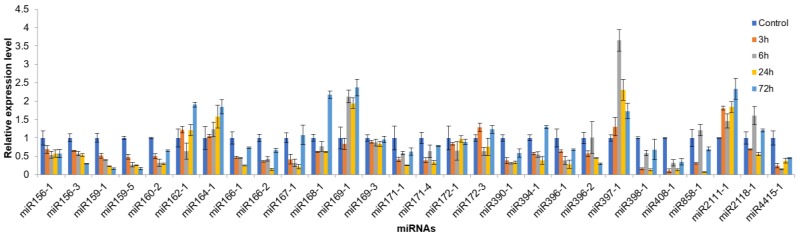
The expression patterns of conserved miRNAs under cold stress in *A. membranaceus* leaves. *A. membranaceus* U6 was used as an internal control. Error bars represent ±SD from three independent experiments.

**Figure 8:**
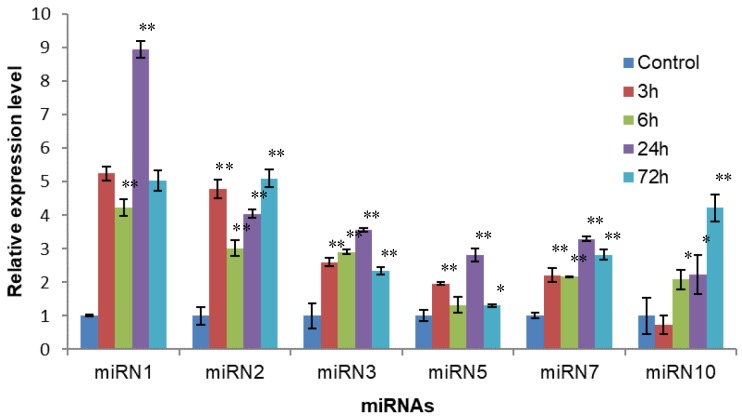
The expression patterns of six selected non-conserved miRNAs under cold stress in *A. membranaceus* leaves. *A. membranaceus* U6 was used as an internal control. Error bars represent ±SD from three independent experiments. * *P* < 0.05 compared to the control group, ** *P* < 0.01 compared to the control group.

As expected, miRNAs from the same family showed similar expression pattern under cold stress. Out of the 28 miRNAs selected from 21 miRNA families, miR168-1, miR169-1, miR397-1, and miR2111-1 were up-regulated at least one time-point during the 72 h cold stress treatment, while most of the remaining miRNAs were down-regulated at least one time-point under cold stress. These down-regulated miRNAs included miR156-3, miR159-1, miR159-5, miR160-2, miR166-1, miR166-2, miR167-1, miR171-1, miR171-4, miR390-1, miR394-1, miR396-1, miR396-2, miR398-1, miR408-1, miR858-1, and miR4415-1 (Figure 7), while all the six randomly selected non-conserved miRNAs were up-regulated under cold stress (Figure 8).

### 3.8. Expression of miRNA Targets in Response to Cold Stress in A. Membranaceus

**Figure 9:**
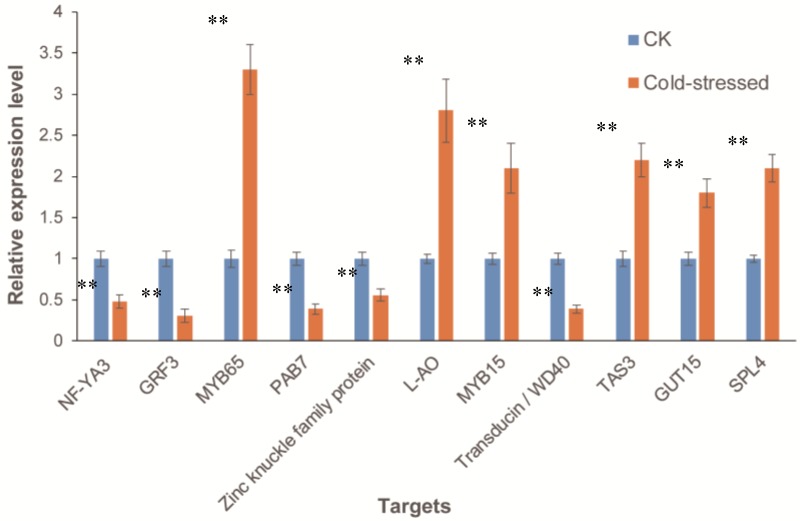
The expression patterns of selected targets of cold-responsive miRNAs under cold stress in *A. membranaceus* leaves. *A. membranaceus* 18S rRNA was used as an internal control. Error bars represent ±SD from three independent experiments. * *P* < 0.05 compared to the control group, ** *P* < 0.01 compared to the control group.

To investigate whether the expressions of the predicted targets were negatively-correlated with that of the corresponding miRNAs, the expression levels of 11 targets of nine cold-responsive miRNAs were further examined by qRT-PCR in *A. membranaceus* leaves after 24 h cold treatment (Figure 9). These 11 targets included several known targets of miR156-3, miR159-1, miR169-1, miR390-1, miR396-1, miR858-1, and miR4415-1, i.e., *squamosa promoter binding protein-like 4* (*SPL4*), *MYB65*, *NF-YA3*, *TAS3*, *GRF3*, *MYB15*, and *L-ascorbate oxidase* (*L-AO*), a novel target of miR156-3, *GUT15*, and 3 randomly selected targets of miR2111-1 and miRN2, *poly(A) binding protein 7* (*PAB7*), *zinc knuckle family protein*, and *transducin*/*WD40*. The results showed that an opposite expression pattern was observed for most of these miRNA-target pairs. *NF-YA3*, *PAB7*, *zinc knuckle family protein*, and *transducin/WD40*, the 4 targets of the three cold-induced miRNAs, miR169-1, miR2111-1, and miRN2, were all down-regulated by cold stress treatment. *TAS3*, *GUT15, SPL4*, *MYB15*, *L-AO*, and *MYB65*, the targets of five cold-repressed miRNAs, miR390-1, miR156-3, miR858-1, miR4415-1, and miR159-1 were up-regulated by cold stress treatment. However, no negative correlations between miR396-1 and its target, *GRF3*, were observed.

## 4. Discussion

Cold stress is one of the major environmental factors affecting the growth, yield, and quality of *A. membranaceus*. miRNAs, a class of endogenous regulatory RNA molecules, may play essential roles in plant cold response. To identify miRNA in *A. membranaceus*, the small RNA extracted from leaves and roots of this plant species was sequenced using the Illumina sequencing platform in the present study. The pattern of sRNA size distribution in *A. membranaceus* is consistent with the typical sRNA length distribution of angiosperms; e.g., *A. thaliana* [18], *Oryza sativa* [43], *Medicago truncatula* [44]), and *Citrus trifoliata* [45] where it has been reported that 24-nt sRNAs dominate the sRNA population followed by 21-nt sRNAs, which falls in the representative size range of Dicer-like (DCL) cleavage products [46].

The miRNA and their targets were identified in *A. membranaceus* for the first time in the present study. A large number of conserved miRNAs distributed in 34 families and 14 non-conserved miRNAs were identified. The majority of the miRNA families identified in *Glycine max* were also found in *A. membranaceus*, indicating the effectiveness of our workflow. However, miRNAs identified in this study might only represent part of miRNAs in *A. membranaceus*. Since the small RNA library was constructed from leaves and roots of seedlings grown under normal conditions, some tissue-specific and stress-induced miRNAs might be missed.

Considering most of the plant miRNA targets have extensive complementarity to their cognate miRNA mature sequences, the miRNA target prediction software like psRNATarget can predict miRNA targets accurately. In the present study, we predicted the miRNA targets in *A. membranaceus* using psRNATarget, and GO analyses of the targets indicated miRNAs participated in various biological processes via negatively regulating many protein-coding targets. In addition, 2 non-coding targets were also found, including *TAS3*, which was targeted by miR390, and *GUT15*, which was probably targeted by miR156-1 in *A. membranaceus*. In many previously studies of miRNA target prediction, only protein-coding targets were identified, thus more attention should be paid to non-coding targets of miRNA in future studies. Indeed, some recent studies have reported that there are many competitive endogenous RNAs (ceRNAs) in cells which can function as sponges for miRNAs through their binding sites, and changes in ceRNA abundances can affect the activity of the corresponding miRNAs [47].

Among the cold-responsive miRNAs identified in the present study, miR156, miR159, miR160, miR166, miR167, miR168, miR169, miR171, miR396, miR397, miR398, and miR408 have been observed to be regulated by various environmental stresses in many plant species [48], while no studies have reported the involvement of miR390, miR394, miR858, and miR2111 in cold response.

It is noteworthy that some development related miRNAs are regulated by cold stress in *A. membranaceus*. miR156 controls developmental timing by targeting *SPL* in *Arabidopsis* [49]. miR156 was observed to be down-regulated under cold stress in rice and *OsmiR156k* overexpression decreased cold tolerance at the growth stage of rice [50]. In the present study, the down-regulation of miR156 in cold stressed *A. membranaceus* may contribute to the cold tolerance by targeting SPL transcription factor genes, such as *SPL3* (*comp19805_c0_seq1*), *SPL4* (*comp24473_c0_seq1*), *SPL12* (*comp22720_c1_seq19*), *SPL13* (*comp17180_c0_seq1*), and *SPL14* (*comp22720_c1_seq5*). miR159 plays an important role in plant development by targeting *MYB* and *TCP* transcription factor genes. miR159 has also been reported to respond to various environmental stresses, and transgenic plants overexpressing miR159 were more sensitive to heat stress in comparison with the wild-type controls in rice, suggesting that down-regulation of miR159 may help to tolerate heat stress [51]. In the present study, the down-regulation of miR159 may participate in the cold stress-induced gene network by activating *ATMYB65*, *TCP3* and *TCP24* homologs in *A. membranaceus*.

miR166 regulates post-transcriptionally the expression of class-III homeodomain-leucine zipper (*HD*-*Zip III*) transcription factor genes, which are critical for root development and axillary meristem initiation [52]. In this study, miR166 was down-regulated by cold treatments in cold stressed *A. membranaceus*, which may target an *ATHB9* homolog (*comp62227_c0_seq1*) and an ATHB15 homolog (*comp21461_c0_seq4*) to mediate the cold induced change in growth and development of *A. membranaceus*.

Plant hormones play key roles in plant adaption to environmental cues by regulating plant growth and development, and auxin and other hormones are involved in plant response to different abiotic stresses [53]. In the present study, several miRNAs that function in auxin were found to be responsive to cold stress in *A. membranaceus*, including miR160, miR167, and miR390. miR167 has been demonstrated to target *ARF6* and *ARF8* in Arabidopsis. In *A. membranaceus*, miR160 was predicted to target *ARF10* and *ARF17* homologs. miR390 and TAS3-derived tasiRNAs form a regulatory module that affect leaf patterning and control lateral root growth by targeting the *ARF* family members *ARF2*, *ARF3* and *ARF4* [54]. In the present study, an additional *CCD8* was predicted to be targeted by *TAS3*-derived tasiRNAs, raising the possibility that miR390 may regulate strigolactone synthesis in *A. membranaceus* under cold stress.

The predicted targets of ame-miR4415 included a plant *L-AO* gene. miR4415 has been reported to be induced by aluminum treatment and drought stress, but down-regulated by *Phakopsora pachyrhizi* infection in soybean [55,56]. L-AO is an apoplastic enzyme that catalyzes the oxidation of ascorbic acid (AA) and plays a vital role in controlling the redox state of the apoplast. Reduction in AA redox state in *L-AO* overexpressed tobacco plants resulted in higher levels of endogenous H_2_O_2_, which enhance the plant tolerance for oxidative stress [57]. miR4415 probably plays a role in controlling the redox state of the apoplast by negatively regulating the expression of *L-AO*. In the present study, down-regulated level of miR4415 might help to cope with oxidative stress imposed by cold stress by activating the expression of *L-AO*.

Previous studies showed that miR858 plays a key role in regulating phenylpropanoid pathway and development via targeting multiple *R2R3 MYB* transcription factor genes. In our study, several miR858 targets were predicted, including 2 *MYB* genes, i.e., *MYB15* and *MYB17*. *MYB15* is required for the defense-induced synthesis of guaiacyl lignin and the basal synthesis of scopoletin, both of which were derived from phenylpropanoid pathway in Arabidopsis [58]. Phosphorylation of the *MYB15* by mitogen-activated protein kinase 6 is necessary for freezing tolerance in *Arabidopsis*, highlighting its important role in cold stress signaling [59]. *MYB17* was demonstrated to regulate the meristem identity transition from vegetative growth to flowering [60]. In cold stressed *A. membranaceus* leaves, the down-regulation of miR858 may modulate phenylpropanoid pathway and meristem identity transition by targeting *MYB15* and *MYB17*, respectively. Considering that environmental factors like cold and drought may affect the phenylpropanoid pathway-derived pharmacological active ingredients [7], we speculate the cold stress-repressed miR858 might affect the accumulation of flavonoids pharmacological active ingredients in *A. membranaceus,* although there is still some research work to be done to determine the link between miR858, cold stress, and flavonoids pharmacological active ingredients.

miR2118 has been demonstrated to target several disease resistant genes in previous studies, and similar targets were predicted for miR2118 in *A. membranaceus* (Table S6). Our results showed that miR2118 was up-regulated after 6 h of cold treatment, and the two targets of miR2118, i.e., a NB-ARC domain-containing disease resistance gene and a TIR-NBS-LRR class disease resistance gene, were down-regulated. Similar expression pattern was observed in leaves of drought-stressed *Caragana intermedia* [61]. These results suggested that miR2118 might participate in cold stress response in *A. membranaceus* by inhibiting expression of disease resistant genes.

miR2111 has been reported to be induced by phosphate starvation but down-regulated under nitrogen deficiency conditions in Arabidopsis [62]. In the present study, miR2111 was up-regulated in leaves after cold treatment. Although many targets, including a gene encoding galactose oxidase/kelch repeat superfamily protein, were predicted to be the targets of miR2111, the exact biological functions of miR2111 in response to environmental stress is still unclear.

Based on our results and related studies, a gene regulation network was constructed to understand the miRNA mediated gene regulation network in responsive to cold stress in *A. membranaceus* (Figure S3). In the network, miR168 plays an important role in miRNA feedback regulation by targeting the *ARGONAUTE* (*AGO*) gene; miR396, miR156, miR159, miR858, miR394, miR160, miR167, miR171, miR166, and miR169 regulate plant grow and development by targeting various transcription factors like SPL, MYB, ARF encoding genes; miR398 and miR4415 contribute to redox homeostasis by targeting *L-AO* and *copper/zinc superoxide dismutase* (*CSD*), respectively; miR397 and miR408 tune lignification of the cell wall by targeting laccase encoding genes. In addition, miR858 might modulate secondary metabolism by targeting *MYB* transcription genes which regulates the phenylpropanoid pathway.

## 5. Conclusions

In summary, in the present study, we employed the high-throughput sequencing technology and bioinformatic approach to identify the conserved and non-conserved miRNAs from *A. membranaceus*, an important medicinal plant. Target prediction of these miRNAs and their functional annotation showed these miRNAs participate in the regulation of various biological processes. Identification of the 2 non-coding targets of miRNAs highlighted the complexity of the miRNA mediated gene regulation network in *A. membranaceus*. Expression analysis of the miRNAs in cold-stressed *A. membranaceus* identified a batch of cold-responsive miRNAs and revealed that miRNAs mediated the response to cold stress by regulating development, hormone signaling, abiotic and biotic stress response, and phenylpropanoid pathway in *A. membranaceus*. The present study will promote understanding of miRNA-mediated post-transcriptional gene regulation in plant response to cold stress. The cold-responsive miRNAs identified may be used as the candidate genes in breeding for improving cold tolerance in *A. membranaceus*.

## Figures and Tables

**Table 1:** Statistical summary of the data that were generated by high-throughput small RNA sequencing in *A. membranaceus*.

Type	Total	Percentage of Total (%)	Distinct	Percentage of Distinct (%)
Raw reads	9,685,427	100.00	3,697,922	100
3ADT & length filter	3,020,110	31.18	949,768	25.68
Junk reads	58,185	0.60	48,416	1.31
Rfam	840,690	8.68	100,245	2.71
Repeats	5584	0.06	1321	0.04
Clean reads	5,761,893	59.49	2,598,545	70.27
rRNA	625,393	6.46	59,659	0.62
tRNA	160,729	1.66	29,496	0.3
snoRNA	5296	0.05	1977	0.02
snRNA	7931	0.08	3860	0.04
Other Rfam RNA	41,341	0.43	5253	0.05

**Table 2:** Predicted conserved miRNAs with identified precursors from *A. membranaceus.*

miRNA Family	miRNA Name	miRNA Sequence (5′-3′)	Length (nt)	Representative Homologous miRNA	Total Reads	Conservation in Other Plant Specie			
						gma	ath	ptc	osa
MIR156	ame-miR156-1	ugacagaagagagugagcac	20	gma-miR156u	13,342	++	+	+	+
	ame-miR156-2	ugacagaagagagagagcac	20	gma-miR156b	10	++	+	+	+
	ame-miR156-3	uugacagaagauagagagcac	21	gma-miR156d	45,036	++	++	++	+
	ame-miR156-4	ugacagaagauagagagcac	20	gma-miR156d	603	+	+	+	+
MIR159	ame-miR159-1	uuuggauugaagggagcucua	21	gma-miR159a-3p	40,206	++	++	++	+
	ame-miR159-2	uuuggauugaagggagcu	18	gma-miR159a-3p	135	++	++	++	++
	ame-miR159-3	uuuggacugaagggagcuccu	21	csi-miR159d	88	+	+	+	+
	ame-miR159-4	uuggacugaaggggccucuu	20	gma-miR319f	48	++	+	+	+
	ame-miR159-5	uuggacugaagggagcuccc	20	ath-miR319a	1459	++	++	++	+
	ame-miR159-6	uggacugaagggagcuccuuc	21	gma-miR319q	437	++	+	+	+
MIR160	ame-miR160-1	ugccuggcucccugaaugcca	21	mtr-miR160c	261	+	+	++	++
	ame-miR160-2	ugccuggcucccuguaugcca	21	gma-miR160a-5p	2374	++	++	++	++
	ame-miR160-3	gcguaugaggagccaagcaua	21	gma-miR160a-3p	127	++	+	+	+
MIR162	ame-miR162-1	ucgauaaaccucugcauccag	21	ath-miR162a-3p	961	++	++	++	++
	ame-miR162-2	ggaggcagcgguucaucgauc	21	csi-miR162-5p	60	+	+	+	+
MIR164	ame-miR164-1	uggagaagcagggcacgugca	21	ath-miR164a	1584	++	++	++	++
	ame-miR164-2	caugugccccucuuccccauc	21	zma-miR164c-3p	41		+	+	
MIR166	ame-miR166-1	ucggaccaggcuucauucccc	21	gma-miR166d	71,955	++	+	+	+
	ame-miR166-2	ucggaccaggcuucauucccg	21	gma-miR166j-3p	8165	++			
	ame-miR166-3	ucucggaccaggcuucauucc	21	gma-miR166k	7665	++	+	+	+
	ame-miR166-4	ggaauguugucuggcucgagg	21	gma-miR166a-5p	276	++	+		+
	ame-miR166-5	ggaauguuguuuggcucgagg	21	gma-miR166h-5p	162	++	+		+
MIR167_1	ame-miR167-1	ugaagcugccagcaugaucuga	22	gma-miR167g	6608	++	+	+	+
	ame-miR167-2	ugaagcugccagcaugaucug	21	gma-miR167c	3720	++	++	++	++
	ame-miR167-3	ugaagcugccagcaugaucua	21	gma-miR167a	2600	++	++	++	++
	ame-miR167-4	gucaugcugugacagccucacu	22	cas-miR167b	210				+
	ame-miR167-5	agaucauguggcaguuucacc	21	ahy-miR167-3p	68			++	+
MIR168	ame-miR168-1	cccgccuugcaucaacugaau	21	aly-miR168a-3p	478		++	++	+
	ame-miR168-2	ucgcuuggugcaggucggga	20	gma-miR168a	30	++	++	++	+
MIR169_2	ame-miR169-1	cagccaaggaugacuugccgg	21	gma-miR169a	105	++	++	++	++
	ame-miR169-2	ugagccagggaugacuugccgg	22	gma-miR169d	12	+	+	+	+
	ame-miR169-3	ugagccaaggaugacuugccgg	22	gma-miR169d	47	++	+	+	+
	ame-miR169-4	ugcagccaaggaugacuugcc	21	gma-miR169b	39	+	+	+	+
	ame-miR169-5	ggcaaguuggccuuggcuau	20	zma-miR169r-3p	4		+	+	+
MIR171	ame-miR171-1	ugauugagccgugccaauauc	21	gma-miR171e	1419	++	+	++	++
	ame-miR171-2	uugagccgcgccaauaucacu	21	gma-miR171k-3p	439	++	+	+	+
	ame-miR171-3	uugagccgugccaauaucac	20	gma-miR171i-3p	87	+	+	+	+
	ame-miR171-4	uugagccgcgucaauaucuca	21	gma-miR171m	1713	++	+	+	+
	ame-miR171-5	agguauuggcgcgccucaauu	21	osa-miR171i-5p	5	+	+	+	+
	ame-miR171-6	cgauguuggugagguucaauc	21	gma-miR171k-5p	40	++	+	+	+
MIR172	ame-miR172-1	agaaucuugaugaugcugcau	21	gma-miR172a	1122	++	++	++	++
	ame-miR172-2	ggagcaucaucaagauucaca	21	aly-miR172c-5p	5	+	+	++	+
	ame-miR172-3	agaaucuugaugaugcugcag	21	ath-miR172c	133	+	++	+	+
	ame-miR172-4	gcagcaucaucaagauucaca	21	csi-miR172b-5p	6	++	+	+	+
MIR390	ame-miR390-1	aagcucaggagggauagcgcc	21	gma-miR390a-5p	350	++	++	++	++
	ame-miR390-2	cgcuauccauccugaguuuca	21	gma-miR390a-3p	11	++	++	+	+
MIR394	ame-miR394-1	uuggcauucuguccaccucc	20	gma-miR394c-5p	546	++	++	++	++
MIR396	ame-miR396-1	uuccacagcuuucuugaacuu	21	gma-miR396b-5p	6105	++	++	++	++
	ame-miR396-2	guucaauaaagcugugggaag	21	gma-miR396i-3p	1717	++	++	+	++
	ame-miR396-3	cucaagaaagcugugggaga	20	gma-miR396b-3p	1313	+	+	+	+
	ame-miR396-4	cuuccacagcuuucuugaacug	22	gma-miR396a-5p	523	+	+	+	+
MIR397	ame-miR397-1	ucauugagugcagcguugaug	21	gma-miR397a	579	++	++	++	++
MIR398	ame-miR398-1	uguguucucaggucaccccuu	21	gma-miR398a	3826	++	++	++	++
	ame-miR398-2	gggucguccugagaccacaug	21	bra-miR398-5p	13	+		+	+
	ame-miR398-3	uguguucucaggucgccccug	21	gma-miR398c	666	++	+	++	++
	ame-miR398-4	ggagugaaucugagaacacaag	22	gma-miR398b-5p	440	+		+	
MIR408	ame-miR408-1	augcacugccucuucccuggc	21	gma-miR408a-3p	3496	++	++	++	+
MIR477	ame-miR477-1	ucccucaaaggcuuccaguau	21	ppt-miR477c	31	+		+	
MIR530	ame-miR530-1	ucugcauuugcaccugcacuu	21	stu-miR530	49	+		+	+
MIR858	ame-miR858-1	cucguugucuguucgaccuug	21	csi-miR858-3p	15		+		
MIR1514	ame-miR1514-1	uuuucauuuuuaaaauaggca	21	gma-miR408a-3p	10	+			
MIR2111	ame-miR2111-1	uaaucugcauccugagguuu	20	gma-miR2111b	197	++	+	+	
	ame-miR2111-2	uaaucugcauccugagguu	19	gma-miR2111b	14	+	+	+	
	ame-miR2111-3	uaaucugcauccugaggugu	20	gma-miR2111b	95	+	+	+	
	ame-miR2111-4	guccuugggaugcagauuacc	21	gma-miR2111a	82	+	+	+	
MIR2118	ame-miR2118-1	uuaccgauuccacccaugccuc	21	mtr-miR2118	850		+	+	+
MIR4415	ame-miR4415-1	uugauucucaucacaacuugg	21	gma-miR4415a-3p	1879	+			
	ame-miR4415-2	auguugugaugggaaucaaug	21	gma-miR4415b-5p	50	+			
MIR5083	ame-miR5083-1	agacuacaauuaucugaucaau	22	osa-miR5083	15				+

The abbreviations represent: gma, Glycine max; ath, Arabidopsis thaliana; ptc, Populus trichocarpa; osa, Oryza sativa; csi, Citrus sinensis; mtr, Medicago truncatula; zma, Zea mays; csa, Camelina sativa; ahy, Arachis hypogaea; aly, Arabidopsis lyrate; bra, Brassica rapa; ppt, Physcomitrella patens; stu, Solanum tuberosum. The plus symbols indicate: ++, miRNA sequences of *A. membranaceus* were exactly identical to those in other species; +, miRNA sequences of *A. membranaceus* were conserved in other species but have variations in some nucleotide positions.

**Table 3:** Non-conserved miRNAs predicted from *A. membranaceus.*

miRNA Name	miRNA Sequence	Length (nt)	Total Reads
ame-miRN-1	guucaagucccuccuuccgc	20	31
ame-miRN-2	uccguuguagucuaguugguc	21	2043
ame-miRN-3	gucgauauguccgagugguuaag	23	1399
ame-miRN-4	caauuuagucccugaaauugu	21	26
ame-miRN-5	gggcauuuggucuagugguaug	22	696
ame-miRN-6	gaagcggaugggggccggcg	20	402
ame-miRN-7	guauuguaaguggcagagug	20	307
ame-miRN-8	acaaaucuuugaaaacaggaa	21	8
ame-miRN-9	ccuguuuucaaauauuuguac	21	2
ame-miRN-10	uauaguuuguuugaugguag	20	78
ame-miRN-11	ugaaucucaguggaucguggc	21	69
ame-miRN-12	gcuuccauagcauagugguag	21	21
ame-miRN-13	uucuaauuucuccucccuuuc	21	7
ame-miRN-14	uuuuugguucauuaaauaacu	21	6

## Supplementary Materials

The following are available online at https://www.mdpi.com/2218-273X/9/5/182/s1. Figure S1: GO enrichment analysis of the predicted miRNA targets, Figure S2: Working model for miR390 mediated gene regulation in responding to cold stress in *A. membranaceus*, Figure S3: The miRNA-mediated gene regulation network in response to cold stress in *A. membranaceus* leaves, Table S1: The primers used for qRT-PCR analysis of miRNAs, Table S2: The primers for qRT-PCR of miRNA targets, Table S3: Predicted conserved miRNAs without identified precursors from *A. membranaceus*, Table S4: The stem-loop precursors of the predicted conserved miRNAs. Table S5: The stem-loop precursors of the predicted non-conserved miRNAs, Table S6: The predicted targets of conserved miRNAs, Table S7: The predicted targets of the non-conserved miRNAs.
