# Supplementary material for: Identification of miRNAs and Their Response to Cold Stress in Astragalus Membranaceus
Source: Biomolecules. 2019 May 10;9(5):182. doi: 10.3390/biom9050182 (PMC6572118; doi:10.3390/biom9050182)
Supplement: Supplementary file 1 [file biomolecules-09-00182-s001.zip › Table S5.docx]

**Table S5 The stem-loop precursors of the predicted non-conserved miRNAs from *A. membranaceus*. The miRNA mature sequences were shown in capital.**

| **miRNA name** | **Stem-loop precursor sequence** | **Length (nt)** | **dG (kcal/mol)** | **MFE/nucleotide (kcal/mol/nt)** |
| --- | --- | --- | --- | --- |
| ame-miRN1 | tagcggaagtgaggtttttaaatgatcatagccttgccaaggctgaggttgaggGTTCAAGTCCCTCCTTCCGCtc | 76 | -33.30 | -0.44 |
| ame-miRN2 | aatatcatctTCCGTTGTAGTCTAGTTGGTCaggatactcggctctcacccgagagacccgggttcaagtcccggcaacggaaattctttttaattattaaagctttgtttttcatttacagctcagaaatgcaaagagaagaaaagcgaaccagaaaaggagtagaaggtgccctgagtgagattatagaggaggagtgaagacg | 206 | -54.43 | -0.26 |
| ame-miRN3 | aGTCGATATGTCCGAGTGGTTAAGgagacagacttgaaatctgttgggctacgcccgcgcaggttcgaaccctgctgtcgacgacttttattaatattttggcctagctgaaaattattgtttggactagcttaacaaaatcataggtaaatttatctgactgtaactataattttaatgaaaactttaattactttttatttcgatg | 208 | -52.60 | -0.25 |
| ame-miRN4 | ttgggtcggtCAATTTAGTCCCTGAAATTGTtcgacatggactattgtggttcagaattttctttttcagggactaaattgaccccacccaat | 93 | -46.60 | -0.50 |
| ame-miRN5 | ggggtcatttattaaccggtgtagtagtaaaataacaataagcatcaggggttgcatgtgaatgcttgtgatgaaactatgttgtatatttgtcggaagaacatgttcaaagaagactgttgtgagtcatagttaactatataaatcttaacctttctttcttgaataGGGCATTTGGTCTAGTGGTATGtcatcagaaa | 200 | -36.42 | -0.18 |
| ame-miRN6 | tgggcttgggGAAGCGGATGGGGGCCGGCGacgtgccctggctgtatgcggaacggctcctgctggtccgccgctcggcttggggcatggactgttgcctgctgcgttggcgtccaaagcacgggg | 119 | -61.60 | -0.49 |
| ame-miRN7 | tccctcttgtccctttataggttgtcatgctggattttttcatggctgaccaactttgtagtcaggtactattttgttggaatctctttcttagtttggttcccttaccaataaaacttaataagagttaaatacatgataaGTATTGTAAGTGGCAGAGTGgc | 164 | -38.80 | -0.24 |
| ame-miRN8 | tttagtttatttCCTGTTTTCAAATATTTGTACaataaaaagtgaaaaaaaattgtttccactgttacctgtACAAATCTTTGAAAACAGGAAacaaagtgaa | 103 | -35.80 | -0.35 |
| ame-miRN9 | tttagtttatttCCTGTTTTCAAATATTTGTACaataaaaagtgaaaaaaaattgtttccactgttacctgtACAAATCTTTGAAAACAGGAAacaaagtgaa | 103 | -35.80 | -0.35 |
| ame-miRN10 | gatcataccattggtcaaaagtatcataactggagcagataaggtatgcaatagatttgaaatttgaattattcaatgaagtttcttttgtaggtgtgattatctggaaaattttctggttaaactgcagtttgtagttctagcatatttattgtttactaagtgatttaggaagctaaaagagttaggaTATAGTTTGTTTGATGGTAGaca | 213 | -50.20 | -0.24 |
| ame-miRN11 | tgattgtaaatacgtcccttgagcttcaattctaagctgcaaatgtttctgcacctgaaaatcatcatcaagatttattagcatgtccttataagttttaatcataatatgttcttcacatgagccttttaggttaaggagaaattgaagtgacaagagcTGAATCTCAGTGGATCGTGGCaataagatca | 191 | -43.12 | -0.23 |
| ame-miRN12 | caatgcatctGCTTCCATAGCATAGTGGTAgactgcagtgtacgtgcagaagtcgatattgtttctcggtctcggagttggggagatgcctaagagccatgccttagtcaggagcttccgccgagaaagcactaaggatccgcttaccctgccctcttcacattctatacatgaggttttgtattcttt | 189 | -50.00 | -0.26 |
| ame-miRN13 | gggaatggaagataagagcagttagaagttgtgggaatgggatggttggaaagaaattatcactgctttgtgttttctttccaattccgcccatgcctactatTTCTAATTTCTCCTCCCTTTCcc | 126 | -54.50 | -0.43 |
| ame-miRN14 | gacaatgcagTTTTTGGTTCATTAAATAACTaatgtaatgactgatgtctccggtctatagatacataagctattcaatgaaacaaaaaactgcattgtctc | 102 | -38.30 | -0.38 |
