# Supplementary material for: Identification of miRNAs and Their Response to Cold Stress in Astragalus Membranaceus
Source: Biomolecules. 2019 May 10;9(5):182. doi: 10.3390/biom9050182 (PMC6572118; doi:10.3390/biom9050182)
Supplement: Supplementary file 1 [file biomolecules-09-00182-s001.zip › Fig_S1_S2_S3.docx]

Supplementary Figures

**Figure S1.** **GO enrichment analysis of the predicted miRNA targets.** (a) Biological process, (b) Cellular component, (c) Molecular function.

**Figure S2.** **The hypothetic** **model for miR390 mediated gene regulation in responding to cold stress in *A. membranaceus*.** The red arrow indicates an up-regulated expression pattern and the green arrow indicates a down-regulated expression pattern.

**Figure S3. The miRNA-mediated gene regulation network in response to cold stress in *A. membranaceus* leaves**. The red and green boxes indicate that the miRNAs were up-regulated or down-regulated, respectively.


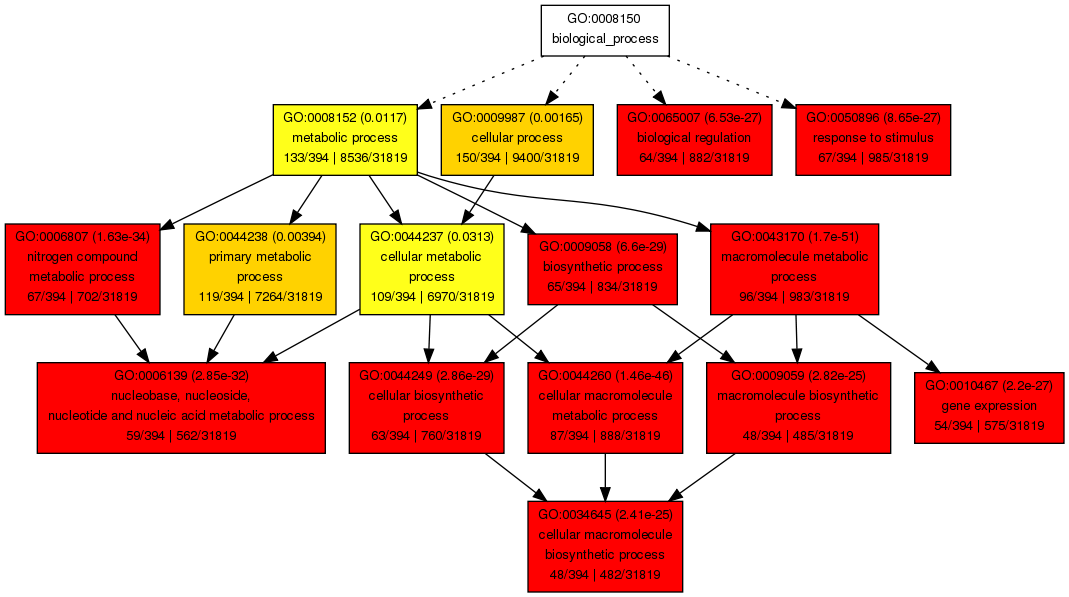


**Figure S1 (a)**


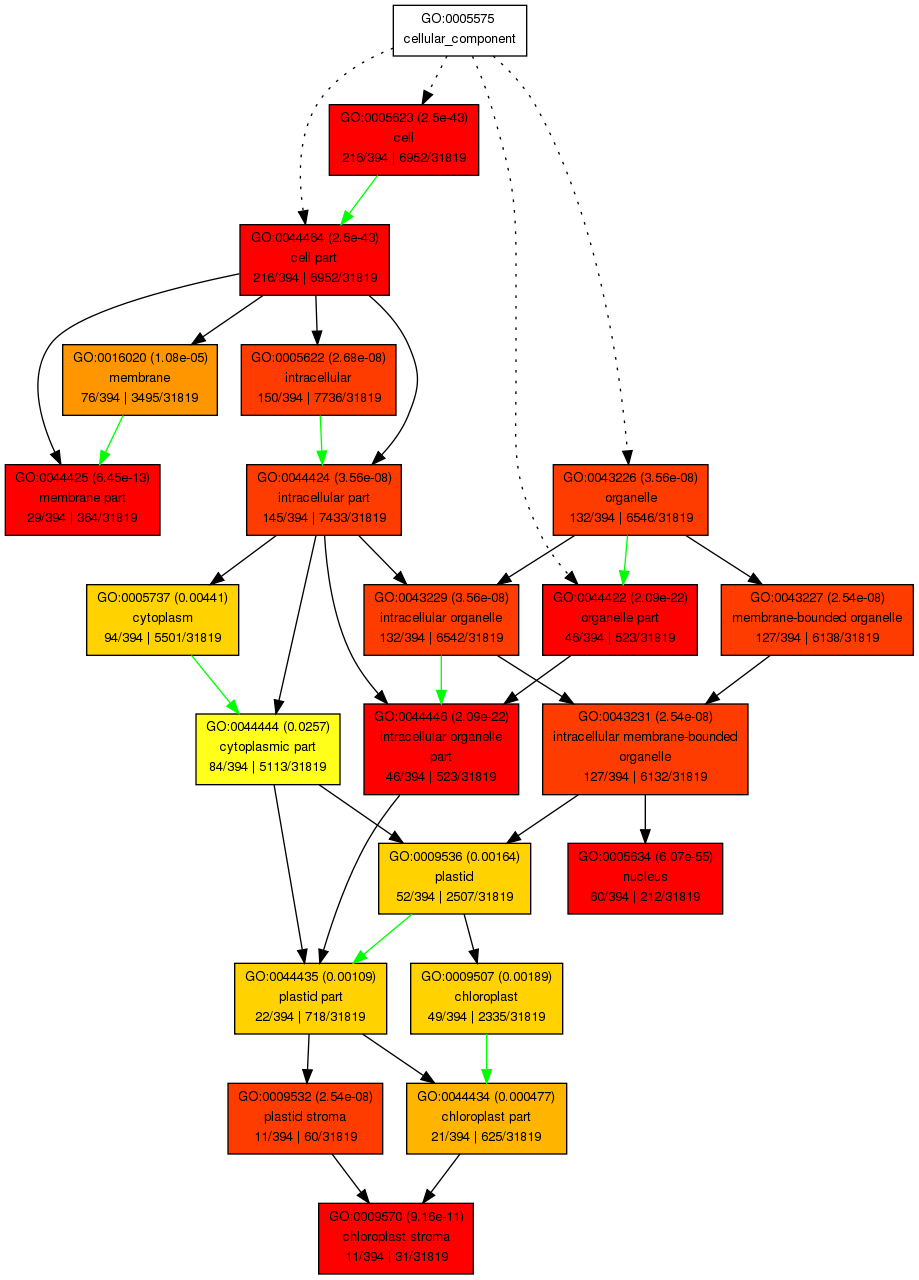


**Figure S1 (b)**


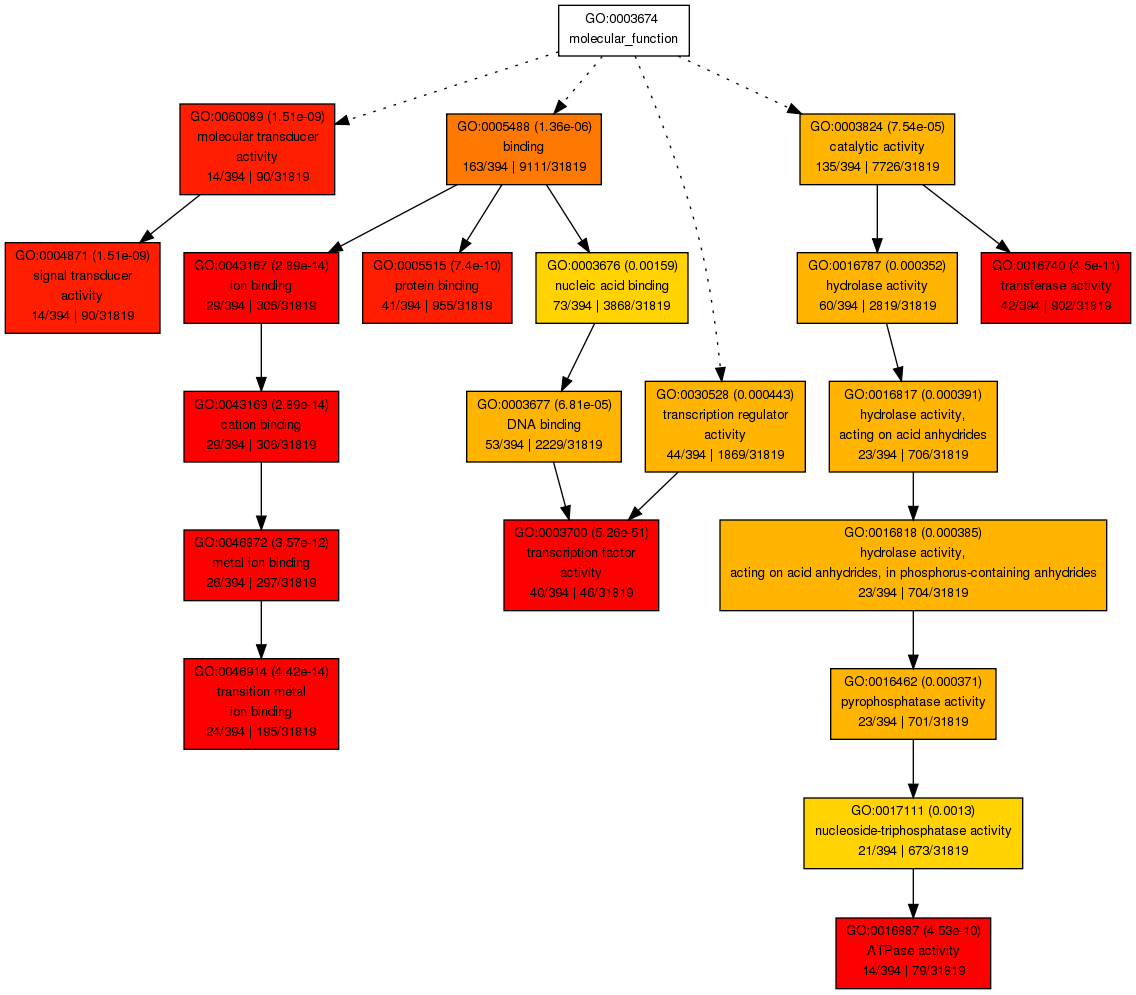


**Figure S1 (c)**

Cold stress

miR390

TAS3 loci

comp6362_c0_seq1

phasiRNAs_2

phasiRNAs_1

ARF2

CCD8

Strigolactone biosynthesis

Auxin signaling

Growth and development

**Figure S2. The hypothetic model for miR390 mediated gene regulation in responding to cold stress in *A. membranaceus*.** The red arrow indicates an up-regulated expression pattern and the green arrow indicates a down-regulated expression pattern.

Cold stressed *A. membranaceus* leaves

Stress perception and signal transduction

miR858

miR159

miR156

miR396

miR2111

miR168

miR390

miR171

miR398

miR4415

miR394

miR160

miR167

miR397

miR166

miR169

miR408

PhasiRNA

SPL

GRF

AGO

MYB

TCP

F-box

LCR

ARF

SCL

HD-ZIP

NF-YA

L-AO

CSD

Laccase

miRNA feedback regulation

Secondary metabolism

Growth and development

Defense

Redox homeostasis

Cell wall lignification

Cold stress response in *A. membranaceus* leaves

**Figure S3. The miRNA-mediated gene regulation network in response to cold stress in *A. membranaceus* leaves**. The red and green boxes indicate that the miRNAs were up-regulated or down-regulated, respectively. F-box, gene encoding a Galactose oxidase/kelch repeat superfamily protein, a target of miR2111.
