# Supplementary material for: Identification of miRNAs and Their Response to Cold Stress in Astragalus Membranaceus
Source: Biomolecules. 2019 May 10;9(5):182. doi: 10.3390/biom9050182 (PMC6572118; doi:10.3390/biom9050182)
Supplement: Supplementary file 1 [file biomolecules-09-00182-s001.zip › Table S1.docx]

**Table S1 The primers used for qRT-PCR analysis of miRNAs.**

| miRNA ID | Forward primer (5'–3') | Reverse primer (5'–3') * |
| --- | --- | --- |
| ame-miR156-1 | gctgacagaagagagtgagcac |  |
| ame-miR159-1 | cgtttggattgaagggtgctct |  |
| ame-miR159-5 | ggttggactgaaggtaactccc |  |
| ame-miR160-1 | cctgtctccctgaatgcca |  |
| ame-miR162-1 | gctcaataaacctcctcatccag |  |
| ame-miR164-1 | tggagaagcagggcacttaca |  |
| ame-miR166-1 | ttgaccaggcttcattcccc |  |
| ame-miR167-1 | gtgaagttgccaggatgttctga |  |
| ame-miR169-4 | cagccacggatgacttgcc |  |
| ame-miR171-1 | gactgagccgtgccaacatc |  |
| ame-miR172-1 | cggagaatcttgatgatgctgtat |  |
| ame-miR390-1 | gaatctcaggagggataacgcc |  |
| ame-miR393-2 | tccaaaggtatcgcattgatcc |  |
| ame-miR394-1 | ttggcattctgtccacctcc |  |
| ame-miR396-1 | gcgttccacagctttcttgaactt |  |
| ame-miR397-1 | gtcattgagagcagcgttgatg |  |
| ame-miR398-1 | ttgtgttctcaggtcacccctt |  |
| ame-miR408-1 | attcactgcctcttccctggc |  |
| ame-miR858-1 | tctcgttgtgtgttcgaccttg |  |
| ame-miR2111-1 | ggtaatccgcatcctgaggttt |  |
| ame-miRN1 | gcgccguucaagucccuccuuccgc |  |
| ame-miRN2 | gcgtccgttgtagtctagttggtc |  |
| ame-miRN3 | gcgtcgatatgtccgagtggttaa |  |
| ame-miRN5 | gggcatttggtctagtggtatg |  |
| ame-miRN7 | ggtattgtaagtggcagagtg |  |
| ame-miRN10 | gcctatagtttgtttgatggtag |  |
| U6 | ggagaagattagcattgcccct | gagaagattagcatggcccct |

*The reverse primer for miRNAs is the universal reverse primer provided in miRcute miRNA first-strand cDNA synthesis kit (Tiangen)
