# Supplementary material for: Identification of miRNAs and Their Response to Cold Stress in Astragalus Membranaceus
Source: Biomolecules. 2019 May 10;9(5):182. doi: 10.3390/biom9050182 (PMC6572118; doi:10.3390/biom9050182)
Supplement: Supplementary file 1 [file biomolecules-09-00182-s001.zip › Table S2 .docx]

Table S2 Primers used for qRT-PCR analyses of miRNA targets.

| miRNAs | Targets | Forward primers | Reverse primers |
| --- | --- | --- | --- |
| miR169-3 | NF-YA3 | agtggtggtctcacctttgg | attgcactgcactgctgaac |
| miR396-1 | GRF3 | ccgctacgagctctcagtct | gaatatcccgggttggtctt |
| miR159-1 | MYB65 | ccgttcaggcctttgatggt | atgcacaggacatggctcat |
| miRN2 | Transducin/WD40 | agacagatcaacggcaccag | ggcgtgtatcaaaggggtca |
| miR2118-1 | NB-ARC domain-containing disease resistance protein | agaaggattgtctgcgccta | tggtaagcatgaccatcgaa |
|  | Disease resistance protein (TIR-NBS-LRR class) family | gccagtttccttgcaaatgt | gacttccaggaccaaaccaa |
| miR2111-1 | PAB7, poly(A) binding protein 7 | aaaacctcggggtgtagctt | agcacctttcatggttccac |
|  | Zinc knuckle (CCHC-type) family protein | ctagtcttccgggctcactg | gaccaatctggaccctgaaa |
| miR858-1 | MYB15, myb domain protein 15 | tgtcgacacctccaaatcaa | cttccgaccaaaaatcctca |
| miR156-1 | GUT15 | cctcgtgtgatgtgtgctct | ctggctatggagttggtggt |
|  | Squamosa promoter binding protein-like 4 (SPL4) | ccaaggctcattccgtactca | acgcctctcattgtgtccag |
| miR390-1 | TAS3 | ccccttcctctctcctcaat | ccaatttcagagccaccaat |
| miR4415-1 | L-ascorbate oxidase | ggttggactgcattgaggtt | cagcctcagcaaaaatgaca |
|  | 18S | tcaaccataaacgatgccgacc | caatccttactatgtctggacctgg |
