# Supplementary material for: Identification of miRNAs and Their Response to Cold Stress in Astragalus Membranaceus
Source: Biomolecules. 2019 May 10;9(5):182. doi: 10.3390/biom9050182 (PMC6572118; doi:10.3390/biom9050182)
Supplement: Supplementary file 1 [file biomolecules-09-00182-s001.zip › Table S3.docx]

Table S3 Predicted conserved miRNAs without identified precursors from *A. membranaceus*.

| Family | miRNA name | miRNA sequence (5′-3′) | Length (nt) | Representative homologous miRNA | Read number |
| --- | --- | --- | --- | --- | --- |
| MIR156 | ame-miR156-5 | tgacagaagagagtgagcaca | 21 | ssp-miR156 | 91 |
|  | ame-miR156-6 | cgacagaagagagtgagcac | 20 | smo-miR156a | 2 |
|  | ame-miR156-7 | tgacagaagagagggagcac | 20 | vvi-miR156a | 24 |
|  | ame-miR156-8 | ctgacagaagatagagagcac | 21 | smo-miR156b | 21 |
|  | ame-miR156-9 | tgtcagaagagagtgagcac | 20 | ghr-miR156c | 3 |
|  | ame-miR156-10 | ttgacagaagaaagagagcac | 21 | smo-miR156c | 2 |
|  | ame-miR156-11 | ttgacagaagagagtgagcac | 21 | sit-miR156d-2 | 146 |
|  | ame-miR156-12 | tgacagaggagagtgagcac | 20 | vvi-miR156e | 5 |
|  | ame-miR156-13 | tgacagaagagagcgagcac | 20 | zma-miR156k | 15 |
|  | ame-miR156-14 | tgacagaagagagtgagcat | 20 | osa-miR156o | 35 |
|  | ame-miR156-15 | tgacagaagagagtgagctc | 20 | osa-miR156p | 2 |
|  | ame-miR156-16 | tgacagaagagagtgggcac | 20 | zma-miR156r | 3 |
|  | ame-miR156-17 | ttgacagaagatagagggcac | 21 | mtr-miR157 | 6 |
|  | ame-miR156-18 | ttgacagaagacagagagcac | 21 | nad-miR157c | 7 |
|  | ame-miR156-19 | ttgacagaagatagagagcat | 21 | sly-miR157c | 158 |
|  | ame-miR156-20 | gctctctatgcttctgtcatc | 21 | aly-miR157d* | 213 |
|  | ame-miR156-21 | ttgacagaagagagagagcac | 21 | zma-miR157m | 80 |
| MIR159 | ame-miR159-7 | tttggactgaagggagctcta | 21 | aqc-miR159 | 4 |
|  | ame-miR159-8 | tttggattgaagggagctctt | 21 | ath-miR159b | 228 |
|  | ame-miR159-9 | attggagtgaagggagctcca | 21 | gma-miR159b | 144 |
|  | ame-miR159-10 | cttggattgaagggagctcta | 21 | osa-miR159f | 15 |
|  | ame-miR159-11 | tttggattgaagggagctctg | 21 | zma-miR159m | 12 |
|  | ame-miR159-12 | agagctttcttcggtccactc | 21 | aly-miR319b* | 3 |
|  | ame-miR159-13 | ttggagtgaagggagctcca | 20 | tae-miR319c | 3 |
|  | ame-miR159-14 | tggactgaaggggagctccttc | 22 | vun-miR319c.2 | 224 |
|  | ame-miR159-15 | ttggactgaaggggcctcttc | 21 | vun-miR319c.4 | 15 |
|  | ame-miR159-16 | ttggactgaagggagctcctt | 21 | rco-miR319d | 37 |
|  | ame-miR159-17 | cttggactgaagggagctccc | 21 | ppt-miR319e | 358 |
|  | ame-miR159-18 | ttggactgaagggagctccct | 21 | vvi-miR319f | 284 |
|  | ame-miR159-19 | ttggactgaagggagctccca | 21 | vvi-miR319g | 6 |
|  | ame-miR159-20 | tgtgaatgatgcgggagctaa | 21 | mtr-miR4414b | 3 |
| MIR160 | ame-miR160-2 | tgcctggctccctgtatgcct | 21 | ttu-miR160b | 7 |
|  | ame-miR160-3 | gcgtatgaggagccaagcata | 21 | gma-miR160a-3p | 127 |
|  | ame-miR160-4 | tgcctggctccctggatgcca | 21 | tcc-miR160c | 4 |
|  | ame-miR160-5 | tgcctggctccctgtatgccg | 21 | zma-miR160f | 3 |
|  | ame-miR160-6 | tgcctggctccctgcatgcca | 21 | ptc-miR160h | 2 |
|  | ame-miR160-7 | tgcctggctccctgtatgcca | 21 | zma-miR160m | 2282 |
| MIR162 | ame-miR162-3 | tggaggcagcggttcatcgatc | 22 | csi-miR162* | 10 |
| MIR164 | ame-miR164-3 | tggagaagcagggcacatgct | 21 | vvi-miR164b | 28 |
|  | ame-miR164-4 | tggagaagcagggcacgtgct | 21 | sit-miR164b | 4 |
| MIR166 | ame-miR166-6 | tcggaccaggcttcattccct | 21 | osa-miR166m | 110 |
|  | ame-miR166-7 | tcggaccaggcttcattccctt | 22 | crt-miR166b | 3 |
|  | ame-miR166-8 | tcggaccaggcttcattcccgt | 22 | csi-miR166b | 9 |
|  | ame-miR166-9 | ctcggaccaggcttcattccc | 21 | bdi-miR166e | 3 |
|  | ame-miR166-10 | tcgaaccaggcttcattcccc | 21 | osa-miR166e | 14 |
|  | ame-miR166-11 | tcggaccaggcttcattcctc | 21 | zma-miR166m | 10 |
|  | ame-miR166-12 | tcggaccaggcttcattcctt | 21 | ptc-miR166q | 3 |
|  | ame-miR166-13 | tcggaccacgcttcattcccc | 21 | zma-miR166u | 22 |
| MIR167_1 | ame-miR167-6 | tgaagctgccagcatgatctta | 22 | mdm-miR167h | 562 |
|  | ame-miR167-7 | tgaagctgccagcatgatctaa | 22 | bna-miR167b | 15 |
| MIR168 | ame-miR168-3 | tcgcttggtgcaggtcgggaa | 21 | vvi-miR168 | 1920 |
| MIR169_2 | ame-miR169-6 | aagccaaggatgacttgccgg | 21 | mtr-miR169l | 61 |
|  | ame-miR169-7 | cagccaaggatgacttgccga | 21 | ath-miR169a-5p | 3 |
| MIR171 | ame-miR171-7 | tattggcctggttcactcaga | 21 | aly-miR171a* | 5 |
|  | ame-miR171-8 | ttgagccgcgccaatatcaca | 21 | ppt-miR171b | 6 |
|  | ame-miR171-9 | tgattgagccgcgtcaatatc | 21 | vvi-miR171b | 591 |
|  | ame-miR171-10 | ttgagccgtgccaatatcacga | 22 | vun-miR171b.3 | 2 |
|  | ame-miR171-11 | agatattggtgcggttcaatc | 21 | gma-miR171c | 35 |
|  | ame-miR171-12 | ttgagccgtgccaatatcaca | 21 | zma-miR171f | 441 |
|  | ame-miR171-13 | tgattgagccgcgccaatatc | 21 | zma-miR171n | 2 |
|  | ame-miR171-14 | tgattgagtcgtgccaatatc | 21 | mtr-miR171 | 2 |
| MIR172 | ame-miR172-5 | agaatcttgatgatgctgca | 20 | zma-miR172d | 21 |
|  | ame-miR172-6 | ggaatcttgatgatgctgcat | 21 | zma-miR172f | 197 |
|  | ame-miR172-7 | agaatcctgatgatgctgcag | 21 | zma-miR172m | 2 |
| MIR390 | ame-miR390-3 | cgctatccatcctgagtttca | 21 | aly-miR390a* | 7 |
|  | ame-miR390-4 | cgctatccatcctgagtttc | 20 | gma-miR390a-3p | 4 |
| MIR393 | ame-miR393-1 | ttccaaagggatcgcattgatc | 22 | vun-miR393b | 1469 |
|  | ame-miR393-2 | tccaaagggatcgcattgatcc | 22 | ath-miR393a-5p | 464 |
|  | ame-miR393-3 | tccaaagggatcgcattgatc | 21 | zma-miR393g | 88 |
|  | ame-miR393-4 | tccaaagggatcgcattgatct | 22 | zma-miR393 | 10 |
| MIR394 | ame-miR394-2 | tttggcattctgtccacctcc | 21 | vun-miR394a.3 | 72 |
| MIR395 | ame-miR395-1 | ctgaagtgtttgggggaactc | 21 | vvi-miR395m | 3 |
| MIR396 | ame-miR396-5 | ttccacagctttcttgaactg | 21 | zma-miR396l | 57734 |
|  | ame-miR396-6 | ttccacagctttcttgaacta | 21 | vvi-miR396a | 329 |
|  | ame-miR396-7 | ttccacggctttcttgaactt | 21 | tcc-miR396d | 3 |
|  | ame-miR396-8 | ttccacagctttcttgaactgt | 22 | gma-miR396e | 41 |
|  | ame-miR396-9 | ttccacggctttcttgaactg | 21 | ptc-miR396f | 66 |
|  | ame-miR396-10 | ttccatagctttcttgaactg | 21 | gcl-miR396b | 12 |
|  | ame-miR396-11 | aagaaagctgtgggagaatatggc | 24 | gma-miR396d | 4 |
| MIR397 | ame-miR397-2 | tcattgagtgcagcgttgatgt | 22 | bna-miR397b | 5 |
| MIR399 | ame-miR399-1 | tgccaaaggagatttgccctg | 21 | mtr-miR399h | 5 |
|  | ame-miR399-2 | tgccaaaggagagttgccctg | 21 | zma-miR399h | 18 |
|  | ame-miR399-3 | cgccaaaggagagttgccctg | 21 | vvi-miR399i | 9 |
|  | ame-miR399-4 | tgccaaaggagaattgccctg | 21 | zma-miR399l | 6 |
| MIR482 | ame-miR482-1 | tcttgcctactccacccatgcc | 22 | ghr-miR482b | 5 |
|  | ame-miR482-2 | tcttcccaattccgcccattccta | 24 | gma-miR482 | 48 |
|  | ame-miR482-3 | tcttcccaattccgcccattcc | 22 | pvu-miR482 | 243 |
| MIR818 | ame-miR818-1 | cttataattagggacggagggagt | 24 | mtr-miR5205b | 2 |
| MIR828 | ame-miR828-1 | tcttgctcaaatgagtattcca | 22 | vvi-miR828a | 27 |
| MIR1511 | ame-miR1511-1 | aaccaggctctgataccatg | 20 | gma-miR1511 | 146 |
| MIR1514 | ame-miR1514-2 | ttcatttttaaaataggcatt | 21 | gma-miR1514a | 17 |
|  | ame-miR1514-3 | atgcctattttaaaatgaaaa | 21 | gma-miR1514a-3p | 28 |
| MIR1515 | ame-miR1515-1 | tcatttttgcgtgcaatgatcc | 22 | csi-miR1515 | 560 |
| MIR2111 | ame-miR2111-5 | taatctgcatcctgaggttta | 21 | vun-miR2111a.2 | 25 |
|  | ame-miR2111-6 | taatctgtatcctgaggttta | 21 | vun-miR2111a.3 | 3 |
|  | ame-miR2111-7 | taatctgcatcctgaggtgta | 21 | vun-miR2111a.4 | 40 |
| MIR2118 | ame-miR2118-2 | ttgccgattccacccattccta | 22 | pvu-miR2118 | 19256 |
|  | ame-miR2118-3 | ttaccgattccacccattccta | 22 | mtr-miR2118 | 6 |
| MIR4416 | ame-miR4416-1 | ctgggtgagagaaacacgtat | 21 | gma-miR4416c | 23 |
