# Supplementary material for: Identification of miRNAs and Their Response to Cold Stress in Astragalus Membranaceus
Source: Biomolecules. 2019 May 10;9(5):182. doi: 10.3390/biom9050182 (PMC6572118; doi:10.3390/biom9050182)
Supplement: Supplementary file 1 [file biomolecules-09-00182-s001.zip › Table S4.docx]

**Table S4 The stem-loop precursors of the conserved miRNAs from *A. membranaceus*. The miRNA mature sequences were shown in capital.**

| **Family** | **ID** | **miRNA sequence** | **Stem-loop precursor sequences** | **Homolog of stem-loop sequences in miRBase** | **dG (kcal/mol)** | **MFE/nucleotide (kcal/mol/nt)** |
| --- | --- | --- | --- | --- | --- | --- |
| MIR156 | miR156-1 | ugacagaagagagugagcac | guaagggaggUGACAGAAGAGAGUGAGCACagaugguauuuucuuguaugauguuucauucuugaaacuaucugugcucacucucuaucugucaccccaucaccau | mtr-MIR156b | -59.60 | -0.56 |
|  | miR156-1 | ugacagaagagagugagcac | auacaauuaugaguguuuuuccucuuuagauuucugucaaguuucuuuuccauaucugggaugcauauauguuuauguagguauguacuugguuucuuauuugggacauagaaauUGACAGAAGAGAGUGAGCACacagagguac | tcc-MIR156g | -37.90 | -0.26 |
|  | miR156-1 | ugacagaagagagugagcac | gaagggagggUGACAGAAGAGAGUGAGCACaugcugcuguuuauuguaucagggcauacaauuucgagugcgugcucacuucucuuucugucaucuuucuaauuu | gma-MIR156a | -48.40 | -0.46 |
|  | miR156-1 | ugacagaagagagugagcac | acauagaaauUGACAGAAGAGAGUGAGCACacagagguacaauuaguauacauuuauacuauugcuuuugugugcucacgccucuuucugucauuuuccagugcc | tcc-MIR156g | -51.10 | -0.49 |
|  | miR156-2 | ugacagaagagagagagcac | ugaaguuugugaacuuauuccuaucaucuuuucagaaacuugacucauacuauaauggcuaugguuguuuggaauaacuaaaggacgagccaugugaauucccgacgauuggcuugaagaguuaugagauugagaUGACAGAAGAGAGAGAGCACaaccagcguc | htu-MIR156a | -39.80 | -0.24 |
|  | miR156-3 | uugacagaagauagagagcac | uugUUGACAGAAGAUAGAGAGCACagcugaacauauacaaaaaggcuuuguguuugagcagauuugugcuuucuuuucuucugucaacau | tcc-MIR156f | -45.20 | -0.50 |
|  | miR156-3 | uugacagaagauagagagcac | cucucaacuacuucuucagcagcaaaacuuuauuccucuccaacacuuccuucaaauaccacacuucuuaccuuuguucucucucuugcucuuucuuaugagauauugaaauucuuaguaguaacuuguguauguauucuaucccuuaugaaugugugaaguuccuaaagaggaauaucaagaaugcuugUUGACAGAAGAUAGAGAGCACagcugaacau | tcc-MIR156f | -46.10 | -0.21 |
|  | miR156-4 | ugacagaagauagagagcac | ccaauaucaagcuuucaucuucucagguacgugcugcuggcuauguuuuuuauuugguguuuucucucucauucaugcaucuuaauuaauuaaaucaauuuucucauguuaauuaagaggagaggcauugaugaugcugcUGACAGAAGAUAGAGAGCACagaaaaaaaa | ppe-MIR156f | -39.40 | -0.23 |
| MIR159 | miR-159-1 | uuuggauugaagggagcucua | uugugguguggagcuccuuuuaguccaaaugaagaucugacuguguugauagagcugcuaagcuauggaucccauaguucuacccauuuauaugaucugugugguaguccugcggcuuccauauccugggagcuucaucaucucuuagucuuaucuuuuUUUGGAUUGAAGGGAGCUCUAcacuauauuu | gma-MIR159e | -93.60 | -0.49 |
|  | miR-159-2 | uuuggacugaagggagcuccu | guagaaaugggaguuccuugcagcccaaagcgcugagaaugucuccuucccucugcuguUUUGGACUGAAGGGAGCUCCUaguucuucac | mtr-MIR319c | -39.70 | -0.44 |
|  | miR-159-3 | uuggacugaaggggccucuu | gaguauugaaggggcucacuucaguccaguucgaggcgguggauguggcugggacucagcugcugacucguugguucgaaagcacaccgaucaucuuaaauggugugcuucugauccaacgaugcgggagcugcauucagucgugacugucacgucUUGGACUGAAGGGGCCUCUUcauaucucuu | rco-MIR319b | -100.20 | -0.54 |
|  | miR-159-5 | uuggacugaagggagcuccc | uaggguaagagagcuuucuucaguccacucauggguggcauauaggauucaauuagcugcugacucauucauccaaaugcugaguuaguuaugugaaaauacucagcaaaugagugaaugaugcgggagacaaauugaaucuuaaguuuccuguacUUGGACUGAAGGGAGCUCCCuuuucuuuuc | gma-MIR319a | -80.90 | -0.43 |
|  | miR-159-4 | uggacugaagggagcuccuuc | aagagagugaaggagcuuccuacagcccaaccaugaauaugagaaaauacuacuugguugcuaaauuaacuacuucuuauaucugaagcucucuuuauucuaauuauguuaauauaaugaaauggaagauauuuugcaccauauauauuuuucucuaugguUGGACUGAAGGGAGCUCCUUCuuucuguu | gma-MIR319g | -66.20 | -0.35 |
| MIR160 | miR160-1 | ugccuggcucccugaaugcca | guUGCCUGGCUCCCUGAAUGCCAuauaagaagcuugucaaaaauguugacaacccuuuuacuuggcauugagggagccaugcaagcuagugcuac | mtr-MIR160f | -46.10 | -0.49 |
|  | miR160-2 | ugccuggcucccuguaugcca | uaugcuuaugUGCCUGGCUCCCUGUAUGCCAuuuguagagcucaucgaaacaucaaugaccuuuguggaugGCGUAUGAGGAGCCAAGCAUAuuccaugu | gma-MIR160a | -50.80 | -0.51 |
|  | miR160-3 | gcguaugaggagccaagcaua | uaugcuuaugUGCCUGGCUCCCUGUAUGCCAuuuguagagcucaucgaaacaucaaugaccuuuguggaugGCGUAUGAGGAGCCAAGCAUAuuccaugu | gma-MIR160a | -50.80 | -0.51 |
| MIR162 | miR162-1 | ucgauaaaccucugcauccag | ugaagucacuGGAGGCAGCGGUUCAUCGAUCucuuccugaauuugguuguggaagaacgcaaaacaagaaucggUCGAUAAACCUCUGCAUCCAGcgcucacuuu | gma-MIR162b | -36.50 | -0.35 |
|  | miR162-2 | ggaggcagcgguucaucgauc | ugaagucacuGGAGGCAGCGGUUCAUCGAUCucuuccugaauuugguuguggaagaacgcaaaacaagaaucggUCGAUAAACCUCUGCAUCCAGcgcucacuuu | gma-MIR162b | -36.50 | -0.35 |
| MIR164 | miR164-1 | uggagaagcagggcacgugca | cugaacaagaUGGAGAAGCAGGGCACGUGCAauucuaacucauaaaauaugaaucucuugagaaugaguuagucuuuCAUGUGCCCCUCUUCCCCAUCaugacaacaa | lus-MIR164c | -42.10 | -0.39 |
|  | miR164-2 | caugugccccucuuccccauc | cugaacaagaUGGAGAAGCAGGGCACGUGCAauucuaacucauaaaauaugaaucucuugagaaugaguuagucuuuCAUGUGCCCCUCUUCCCCAUCaugacaacaa | lus-MIR164c | -42.10 | -0.39 |
| MIR166 | miR166-1 | ucggaccaggcuucauucccc | uugguugagaGGAAUGUUGUCUGGCUCGAGGuuauggauggaggagauacugaucauauucucaugaucaguaguguaauugaaauuuacuacccucuaacaaucUCGGACCAGGCUUCAUUCCCCccacccagcu | pvu-MIR166a | -54.30 | -0.40 |
|  | miR166-1 | ucggaccaggcuucauucccc | ucuuuugaggGGAAUGUUGUCUGGCUCGAGGacccuuuucucuuugauccaaaauauagagaauucaagauucuaauucacucguagguuguuguggucaauaaauguuuaguguugUCGGACCAGGCUUCAUUCCCCccaauuguau | ppe-MIR166d | -46.50 | -0.31 |
|  | miR166-3 | ggaauguugucuggcucgagg | ucuuuugaggGGAAUGUUGUCUGGCUCGAGGacccuuuucucuuugauccaaaauauagagaauucaagauucuaauucacucguagguuguuguggucaauaaauguuuaguguugUCGGACCAGGCUUCAUUCCCCccaauuguau | ppe-MIR166d | -46.50 | -0.31 |
|  | miR166-1 | ucggaccaggcuucauucccc | aaaguugaggggaacgucgucuggcucgagaucauucauaaauagacugaucagagacauaacucuugugaaugauuUCGGACCAGGCUUCAUUCCCCucaacaacca | gma-MIR166g | -52.20 | -0.48 |
|  | miR166-4 | ucucggaccaggcuucauucc | gguugaugGGAAUGUUGUUUGGCUCGAGGuugaauaaauaacuucauuauuguuugccauugaagcuagcuagcuucaaagguuugauauugaagaugugaUCUCGGACCAGGCUUCAUUCCcguuaacuug | cme-MIR166g | -59.70 | -0.45 |
|  | miR166-5 | ggaauguuguuuggcucgagg | gguugaugGGAAUGUUGUUUGGCUCGAGGuugaauaaauaacuucauuauuguuugccauugaagcuagcuagcuucaaagguuugauauugaagaugugaUCUCGGACCAGGCUUCAUUCCcguuaacuug | cme-MIR166g | -59.70 | -0.45 |
|  | miR166-1 | ucggaccaggcuucauucccc | auggauuuguggggaaugcuaccugguccaaggagaugaagaugauacuaccacuaccuuucagaaaauuuuguuuaucagauuauucagauuguguuugugaguaguuuaguuauauaugucuccUCGGACCAGGCUUCAUUCCCCucaaguacag | gma-MIR166r | -61.50 | -0.39 |
|  | miR166-5 | ggaauguuguuuggcucgagg | gggguugaugGGAAUGUUGUUUGGCUCGAGGuugaauaaauaacuucauuauuguuugccauugaagcuagcuagcuucaaagguuugauauugaagaugugaucUCGGACCAGGCUUCAUUCCCGuuaacuug | [cme-MIR166g](http://www.mirbase.org/cgi-bin/mirna_entry.pl?acc=MI0023269) | -61.20 | -0.46 |
|  | miR166-2 | ucggaccaggcuucauucccg | gggguugaugGGAAUGUUGUUUGGCUCGAGGuugaauaaauaacuucauuauuguuugccauugaagcuagcuagcuucaaagguuugauauugaagaugugaucUCGGACCAGGCUUCAUUCCCGuuaacuug | [cme-MIR166g](http://www.mirbase.org/cgi-bin/mirna_entry.pl?acc=MI0023269) | -61.20 | -0.46 |
| MIR167 | miR167-1 | ugaagcugccagcaugaucuga | cuuuagcaguUGAAGCUGCCAGCAUGAUCUGAgcuuuaccuucuaauaggaacaacAGAUCAUGUGGCAGUUUCACCuucugauguu | gso-MIR167a | -37.00 | -0.43 |
|  |  | agaucauguggcaguuucacc | cuuuagcaguUGAAGCUGCCAGCAUGAUCUGAgcuuuaccuucuaauaggaacaacAGAUCAUGUGGCAGUUUCACCuucugauguu | gso-MIR167a | -37.00 | -0.43 |
|  |  | ugaagcugccagcaugaucua | aaggaaaaagUGAAGCUGCCAGCAUGAUCUAgcguugguuagagagcugagaaugagauagcaaagugguaacugguaaccuaacuaaccuuuacuagGUCAUGCUGUGACAGCCUCACUcuuuccuauu | mdm-MIR167e | -53.30 | -0.41 |
|  |  | gucaugcugugacagccucacu | aaggaaaaagUGAAGCUGCCAGCAUGAUCUAgcguugguuagagagcugagaaugagauagcaaagugguaacugguaaccuaacuaaccuuuacuagGUCAUGCUGUGACAGCCUCACUcuuuccuauu | mdm-MIR167e | -53.30 | -0.41 |
|  | miR167-1 | ugaagcugccagcaugaucuga | cgacagcaguUGAAGCUGCCAGCAUGAUCUGAgcuuaccuauagcuucuuauauaugugguaugaauagaucaugugggagcuucaccuguugaaugg | lja-MIR167 | -42.10 | -0.43 |
|  |  | ugaagcugccagcaugaucug | uuugagagguUGAAGCUGCCAGCAUGAUCUGguaauuuauaacaugugguuucaucagaucaucuugcagcuucaaucacucaauca | mes-MIR167a | -40.00 | -0.46 |
|  |  | ugaagcugccagcaugaucua | ggUGAAGCUGCCAGCAUGAUCUAgguuugguuacagugagagcuagaaauauugaucgucgaucgagugcuucauaacccuaacuaggucaugcugugcuggccucacuucuuccuauu | ppe-MIR167a | -47.20 | -0.40 |
|  |  | ugaagcugccagcaugaucua | uugUGAAGCUGCCAGCAUGAUCUAgcguugguuagagagcugagaaugagauagcaaagugguaacugguaaccuaacuaaccuuuacuaggucaugcugugacagccucacucuuuccuauu | mdm-MIR167e | -49.20 | -0.40 |
| MIR168 |  | ucgcuuggugcaggucggga | ugucucuaauUCGCUUGGUGCAGGUCGGGAgccguugcugcgguuuucccgcguaaugauaaagcgguugccggcggcgaaaauuggauCCCGCCUUGCAUCAACUGAAUcggaggccac | [gma-MIR168a](http://www.mirbase.org/cgi-bin/mirna_entry.pl?acc=MI0001779) | -59.80 | -0.50 |
|  |  | cccgccuugcaucaacugaau | ugucucuaauUCGCUUGGUGCAGGUCGGGAgccguugcugcgguuuucccgcguaaugauaaagcgguugccggcggcgaaaauuggauCCCGCCUUGCAUCAACUGAAUcggaggccac | [gma-MIR168a](http://www.mirbase.org/cgi-bin/mirna_entry.pl?acc=MI0001779) | -59.80 | -0.50 |
| MIR169 |  | ugcagccaaggaugacuugcc | gauauagugcUGCAGCCAAGGAUGACUUGCCgagaaauucguucuccaacuuaguccaaugauagugauuuguugcagaauuacuuagaaugauaguaauuauuguugugauauuacugauacaauaugcacuacguacacuauugugaaguguguuuugggauaagguaaauuggcaagucuucuuuggcuacaugucuaucucau | gma-MIR169b | -66.00 | -0.32 |
|  |  | ugagccagggaugacuugccgg | agaguagaugUGAGCCAGGGAUGACUUGCCGGcaugcauucugcaugcaugcaucagcuagcuguguugcuggcaguuguccuugguucauguuugcucuau | mtr-MIR169h | -60.50 | -0.59 |
|  |  | ugagccaaggaugacuugccgg | uuuguguguuggaauuucagccuuugacuuuguuuacuuauaaauacauugacauccauaauguccaauagcauagacaaugaaacguaaaaugugaguuuaaugauguugauuuugcucaagugucuugcaugaagagggaaagagaguauaucUGAGCCAAGGAUGACUUGCCGGcauuuugagg | gma-MIR169e | -37.60 | -0.20 |
|  |  | cagccaaggaugacuugccgg | uagaguagugCAGCCAAGGAUGACUUGCCGGcaaguucaagugcuuucuggucacucguuccgccgcgucggcaaguugucuuuggcuauauuagucucuc | mtr-MIR169a | -45.70 | -0.45 |
|  |  | cagccaaggaugacuugccgg | gaggguaaugCAGCCAAGGAUGACUUGCCGGccggccgaaggcguuagugaaggaaaacaaacaucccccaguuggccGGCAAGUUGGCCUUGGCUAUguugggcucuu | mtr-MIR169a | -53.80 | -0.49 |
|  |  | ggcaaguuggccuuggcuau | gaggguaaugCAGCCAAGGAUGACUUGCCGGccggccgaaggcguuagugaaggaaaacaaacaucccccaguuggccGGCAAGUUGGCCUUGGCUAUguugggcucuu | mtr-MIR169a | -53.80 | -0.49 |
|  |  | cagccaaggaugacuugccgg | accauggcugCAGCCAAGGAUGACUUGCCGGaaagcuucuuuuuguaaugugaccaguuauccggcaagucgucucuggcuaaauaaugaccuc | gma-MIR169v | -40.00 | -0.43 |
| MIR171 |  | uugagccgugccaauaucac | gauucaacgGGAUAUUGGUGCGGUUCAAUGagaaagcaaugcucaaaguguaugaggguuugcuuuucgaUUGAGCCGUGCCAAUAUCACgugucacugc | ppe-MIR171a | -47.40 | -0.47 |
|  |  | ggauauuggugcgguucaaug | gauucaacgGGAUAUUGGUGCGGUUCAAUGagaaagcaaugcucaaaguguaugaggguuugcuuuucgaUUGAGCCGUGCCAAUAUCACgugucacugc | ppe-MIR171a | -47.40 | -0.47 |
|  |  | cgauguuggugagguucaauc | augauaaaagCGAUGUUGGUGAGGUUCAAUCcgaagacggauuuacauguagaagcaguaaaauacgaucucagaUUGAGCCGCGCCAAUAUCACUuauaauugcu | gma-MIR171k | -40.10 | -0.38 |
|  |  | uugagccgcgccaauaucacu | augauaaaagCGAUGUUGGUGAGGUUCAAUCcgaagacggauuuacauguagaagcaguaaaauacgaucucagaUUGAGCCGCGCCAAUAUCACUuauaauugcu | gma-MIR171k | -40.10 | -0.38 |
|  |  | ugauugagccgugccaauauc | augaucgagauauuggugcgguucaaucagaaggcagugcuuuuuauaauccaacgaagcaaagcucuguguuauuUGAUUGAGCCGUGCCAAUAUCc | mtr-MIR171f | -43.70 | -0.45 |
|  |  | ugauugagccgugccaauauc | uggaugaagauguuggaacggcucaaucaaaccaaaucucccgcuggugguuggguucuuugauaUGAUUGAGCCGUGCCAAUAUCaauuccuaau | mes-MIR171k | -41.90 | -0.44 |
|  |  | ugauugagccgugccaauauc | uuuaacgagauauuggugcgguucaauaagaaaguaaugcucaaaaauguuuugagcacuguuuuuUGAUUGAGCCGUGCCAAUAUCacgagccacu | gma-MIR171a | -45.20 | -0.47 |
|  |  | uugagccgcgucaauaucuca | caaaaaagcaAGGUAUUGGCGCGCCUCAAUUugaagacaugguuaacaucaaaaccagcaagcuagccauguaguuuaaUUGAGCCGCGUCAAUAUCUCAucuuccacuu | gma-MIR171m | -39.30 | -0.36 |
|  |  | agguauuggcgcgccucaauu | caaaaaagcaAGGUAUUGGCGCGCCUCAAUUugaagacaugguuaacaucaaaaccagcaagcuagccauguaguuuaaUUGAGCCGCGUCAAUAUCUCAucuuccacuu | gma-MIR171m | -39.30 | -0.36 |
| MIR172 |  | ggagcaucaucaagauucaca | guuugccgguGGAGCAUCAUCAAGAUUCACAugauuuauuagaggguuuuuguuugaugugguccuuauugcuucaaauuaauuagcccuuuuuguuauaugggaaucuugaugaugcugcagcagcaauaag | gma-MIR172c | -52.20 | -0.39 |
|  |  | agaaucuugaugaugcugcau | auuugcggauguagcaucaucaagauucacaugcaaaugcacucucugauuggucuuuugauagugAGAAUCUUGAUGAUGCUGCAUcagccacuaa | mdm-MIR172g | -41.90 | -0.43 |
|  |  | agaaucuugaugaugcugcau | uugcugauguagcaucaucaagauucacaugcaaaugcagauggaaauuggaauuugauguaguacuguaaccaaagguuuuaaaaaaagguucgcaaccgcgaucuauaucaauguuuuugauguuucugcgaccacaauuuaaaaccuuagugugagugcuaugccaauccauugauauuuugaaaugAGAAUCUUGAUGAUGCUGCAUuggccauaaa | lja-MIR172b | -83.30 | -0.38 |
|  |  | agaaucuugaugaugcugcau | uuuugaaaugAGAAUCUUGAUGAUGCUGCAUuggccauaaacgacuuuauaaaguugaauaagguaguuuuaccccaacaaguggaaggggcaaagaaaaagagaagugcauaaacguguggauacgguuuaugacacaacugaaagguauuucugcuuugguuuguguugucaucagguggguuacauuuuuuucuu | vun-MIR172 | -49.60 | -0.25 |
|  |  | ggagcaucaucaagauucaca | guuugcugauGGAGCAUCAUCAAGAUUCACAagcuuuagggguuuuuaauuuggagugguuccuaauuauugcuacaaaucuauuugcccuugauaugAGAAUCUUGAUGAUGCUGCAGcggcaauaaa | gma-MIR172d | -55.80 | -0.43 |
|  |  | agaaucuugaugaugcugcag | guuugcugauGGAGCAUCAUCAAGAUUCACAagcuuuagggguuuuuaauuuggagugguuccuaauuauugcuacaaaucuauuugcccuugauaugAGAAUCUUGAUGAUGCUGCAGcggcaauaaa | gma-MIR172d | -55.80 | -0.43 |
|  |  | gcagcaucaucaagauucaca | augugcagguGCAGCAUCAUCAAGAUUCACAucaggcuuccauugaucuuauacauguauauguauguauauguaugaguuuguuggcuuucuugaaguuucauuagagaaaaugugaaucuugaugaugcugcaucagcaaauau | mes-MIR172d | -67.90 | -0.47 |
|  |  | gcagcaucaucaagauucaca | auuugcugauGCAGCAUCAUCAAGAUUCACAuuuucucuaaugaaacuucaagaaagccaacaaacucauacauauacauacauauacauguauaagaucaauggaagccugaugugaaucuugaugaugcugcaccugcacauac | mes-MIR172d | -51.90 | -0.36 |
| MIR390 |  | aagcucaggagggauagcgcc | cauaAAGCUCAGGAGGGAUAGCGCCguuacacuuacauauauaguugagauuggcgcuaucuauccugaguuucauggguucuuc | bna-MIR390b | -34.30 | -0.40 |
|  |  | aagcucaggagggauagcgcc | ugaaucuguaAAGCUCAGGAGGGAUAGCGCCauagaugaucuuuuucuccauucuaaugaucgaucuucucuugCGCUAUCCAUCCUGAGUUUCAuggcuucuuc | lja-MIR390b | -43.00 | -0.41 |
|  |  | cgcuauccauccugaguuuca | ugaaucuguaAAGCUCAGGAGGGAUAGCGCCauagaugaucuuuuucuccauucuaaugaucgaucuucucuugCGCUAUCCAUCCUGAGUUUCAuggcuucuuc | lja-MIR390b | -43.00 | -0.41 |
| MIR394 |  | uuggcauucuguccaccucc | cacagccauuUUGGCAUUCUGUCCACCUCCauuauaauggauaguugacaauuggagguggccaguaggccaaacgagcucugug | gma-MIR394f | -33.80 | -0.40 |
|  |  | uuggcauucuguccaccucc | acagaguuuaUUGGCAUUCUGUCCACCUCCaccuucgaucuaucgaucuuuugcggaggugggcauacugccaacagagcuguguu | gma-MIR394g | -39.40 | -0.46 |
|  |  | uuggcauucuguccaccucc | cagagauuuuUUGGCAUUCUGUCCACCUCCacuuucccaacuacgcuagcuacguacucgcggagguggucauacugccaacugaccucuguu | ptc-MIR394a | -32.20 | -0.35 |
| MIR396 |  | uuccacagcuuucuugaacuu | ggucaugcuuUUCCACAGCUUUCUUGAACUUcuuuugcaacuuaagcuucuuucuccagaauuuauagcccuagaagcucaagaaagcugugggagaauauggcaau | gma-MIR396b | -47.20 | -0.44 |
|  |  | cuuccacagcuuucuugaacug | ucuuuuuauuCUUCCACAGCUUUCUUGAACUGcauacaaagagagaguuucuuucuuucuuuucaugcaugcuauggcuuucuugcuucuacaucuuguuuugcgGUUCAAUAAAGCUGUGGGAAGauacaaauag | gma-MIR396a | -47.90 | -0.35 |
|  |  | guucaauaaagcugugggaag | ucuuuuuauuCUUCCACAGCUUUCUUGAACUGcauacaaagagagaguuucuuucuuucuuuucaugcaugcuauggcuuucuugcuucuacaucuuguuuugcgGUUCAAUAAAGCUGUGGGAAGauacaaauag | gma-MIR396a | -47.90 | -0.35 |
|  |  | cucaagaaagcugugggaga | cccuagaagCUCAAGAAAGCUGUGGGAGAauauggcaauucaggcuuuuugguaauuuaacuccucuugcagcaguugcagacuacacuacacgguucuucucuccugcauccuuaauugucaugguuuaggga | gma-MIR396k | -32.70 | -0.24 |
| MIR397 |  | ucauugagugcagcguugaug | ugaagaaacaUCAUUGAGUGCAGCGUUGAUGaaaauccuaauuauagcaacuaccuugugucucuucacuuauagaugucaaguaguauuuucaucgacgcugcauucaaucauguuuuuuggaa | gma-MIR397b | -52.70 | -0.42 |
| MIR398 |  | gggucguccugagaccacaug | ggguucuacaGGGUCGUCCUGAGACCACAUGaagcuugcucugguuuucacaauacuacaagcuauucauuucaUGUGUUCUCAGGUCGCCCCUGcuguacuuuu | cme-MIR398a | -41.90 | -0.40 |
|  |  | uguguucucaggucgccccug | ggguucuacaGGGUCGUCCUGAGACCACAUGaagcuugcucugguuuucacaauacuacaagcuauucauuucaUGUGUUCUCAGGUCGCCCCUGcuguacuuuu | cme-MIR398a | -41.90 | -0.40 |
|  |  | ggagugaaucugagaacacaag | uuaucucagaGGAGUGAAUCUGAGAACACAAGuuggauuaauuaguugcacagccauaucaucaauugguauaugaucuguuaauugcuaauuuaauuUGUGUUCUCAGGUCACCCCUUugagccaacc | gma-MIR398a | -55.50 | -0.43 |
|  |  | uguguucucaggucaccccuu | uuaucucagaGGAGUGAAUCUGAGAACACAAGuuggauuaauuaguugcacagccauaucaucaauugguauaugaucuguuaauugcuaauuuaauuUGUGUUCUCAGGUCACCCCUUugagccaacc | gma-MIR398a | -55.50 | -0.43 |
| MIR408 |  | augcacugccucuucccuggc | aagacaaagccagggaacagacagagcauggauggaacucucaacagaacagaaacugaugaugauuagaaugagaaugagaggaguaaucugucguguucuacucAUGCACUGCCUCUUCCCUGGCucucuuucua | gma-MIR408a | -49.90 | -0.36 |
| MIR477 |  | ucccucaaaggcuuccaguau | cucuucauguUCCCUCAAAGGCUUCCAGUAUucguggucauauauacuaacuugaauccugguggcuuuugauggaacuugauagcuu | vvi-MIR477a | -32.20 | -0.37 |
| MIR530 |  | ucugcauuugcaccugcacuu | guugccuuuaUCUGCAUUUGCACCUGCACUUuaauuacuugguuauuuucauuaaaacauauuauugucaccgauauguuguagaagggaaccaagacaaggugcaggugcauaugcagaugaaugccauau | ptc-MIR530a | -58.50 | -0.44 |
|  |  | ucugcauuugcaccugcacuu | guugccuuuaUCUGCAUUUGCACCUGCACUUcauuacuugguuuucucgcuucaacuuagacuccacaaaaaauaugcauaucucaauggauaaaaaaauacguauuauauaugugggaguacguagacgagggaccaagcacgaggugcaggugcuucugcagaugaaugccaucu | gma-MIR530c | -69.00 | -0.39 |
| MIR858 |  | cucguugucuguucgaccuug | uaaucuuuauCUCGUUGUCUGUUCGACCUUGacuccuuaucuucacuucaacuuaaaaacaucaaauauuuacugaaguauaucuaauuaauguuuacuacauuucuuuuuguuaaauuaauaaaauaauguugcaccaucucucucuauagaaacuauauuaucucaaggucggaaaggcagcgaauuugaguuccug | ppe-MIR858 | -40.00 | -0.20 |
| MIR1514 |  | uuuucauuuuuaaaauaggca | ccccauucccUUUUCAUUUUUAAAAUAGGCAcugcauugcgaaauccaccgcaccggccucuguagugccuauuuuaaaaugaaaacaaccacgauac | pvu-MIR1514a | -31.50 | -0.32 |
| MIR2111 |  | uaaucugcauccugagguuu | aaggaugggaUAAUCUGCAUCCUGAGGUUUggagcaauauuuuauaguugucucuagcccuugggaugcggauuaucucuuccuuau | ppe-MIR2111b | -51.30 | -0.59 |
|  |  | uaaucugcauccugagguuu | aaggauggggUAAUCUGCAUCCUGAGGUUUagggcauuguaauuuuauuugucucugauccuugggaugcugauuaucucuuccuuau | gma-MIR2111a | -44.50 | -0.51 |
|  |  | uaaucugcauccugagguuu | uaggauuuggUAAUCUGCAUCCUGAGGUUUaaaaacaauagauuuauugucucuaguccuuaggaugcagauuaccucuuccuugu | lja-MIR2111 | -43.00 | -0.50 |
|  |  | uaaucugcauccugagguuu | aaggauaaggUAAUCUGCAUCCUGAGGUUUagggcauuguaauuuuauuugucucugauccuugggaugcugauuaucucuuccuuau | gma-MIR2111a | -42.90 | -0.49 |
|  |  | uaaucugcauccugaggugu | uaggaaugcgUAAUCUGCAUCCUGAGGUGUagagcaauauuuguuuuauuguauccacucuuugggaugcagauuacuucuuccuugu | gma-MIR2111a | -40.10 | -0.46 |
|  |  | guccuugggaugcagauuacc | aaggauuggguaaucugcauccuaagguuuagaauaaugauugaagcauuguaucuaGUCCUUGGGAUGCAGAUUACCuuuuccccau | gma-MIR2111a | -49.40 | -0.56 |
| MIR2118 |  | uuaccgauuccacccaugccu | aggaaauuauggguuaugggaggauugguaaagcagaucuuaguugauucaagcuUUACCGAUUCCACCCAUGCCUcaugauuucca | mtr-MIR2118 | -46.70 | -0.54 |
| MIR4415 |  | auguugugaugggaaucaaug | AUGUUGUGAUGGGAAUCAAUGguagcaaggaucauugccaUUGAUUCUCAUCACAACUUGGuc | gma-MIR4415b | -34.00 | -0.54 |
|  |  | uugauucucaucacaacuugg | AUGUUGUGAUGGGAAUCAAUGguagcaaggaucauugccaUUGAUUCUCAUCACAACUUGGuc | gma-MIR4415b | -34.00 | -0.54 |
| MIR5083 |  | agacuacaauuaucugaucaau | uuauacaaucAGACUACAAUUAUCUGAUCAAUggcucucuccuuaaauaugauuuuucugaugaaguacuauauuuuuugaaguuugaacuacguaagugauugaguuuuaaaaauuugucaauuuacuugauugcuaauuaguuuaucaagaaauuguuuguuauuuuuaucuaaauuuuuagaauuugaucgaugauugaucugaaccaacugaa | gra-MIR8762c | -43.90 | -0.20 |
